# Preparing fertile ground: how does the quality of business environments affect MSE growth?

**DOI:** 10.1007/s11187-023-00804-z

**Published:** 2023-08-16

**Authors:** Jonathan Fu, Annette Krauss

**Affiliations:** Department of Banking and Finance, University of Zurich, Plattenstrasse 32, 8032 Zurich, Switzerland

**Keywords:** Enterprise growth, Microfinance, Business environments, Policy shock, D22, G21, O12, R1

## Abstract

We study how the quality of local business environments helps explain growth outcomes of micro- and small enterprise microfinance clients by drawing on long-term nationwide administrative data and a policy shock in Cambodia. The staggered launch of special economic zones, which we link to positive shocks to the business environment on both the demand and supply side, leads to significantly increased employment in micro- and small enterprises (MSEs) located in these special economic zones (SEZs), compared to enterprises in contextually similar districts that are unexposed to an SEZ. Key channels explaining the improved growth outcomes include expanded access to external markets for the enterprises’ goods and services, more dynamic labor environments, and improved credit terms and conditions. To broaden the relevance of our findings, we combine data from prominent empirical studies on microfinance and demonstrate how related business conditions identified in the enterprise growth literature help explain differences in client business outcomes found in their results. Policy implications are that a smaller but influential segment of microfinance borrowers significantly benefit from opportunities provided by improved local business environments and that governments and lenders can play active roles in facilitating the necessary improvements for such MSEs.

## Abstract (plain language summary)

Financial access may not be fulfilling its real potential in low-income settings, unless coupled with the right opportunities in the business environment for micro- and small enterprises. We use Cambodia’s special economic zones as a policy shock to study how the quality of local business environments help explain micro- and small enterprise microfinance clients’ growth. We find that the SEZs generate large increases in employment for microfinance borrowers for both micro- and small enterprises. These increases are concentrated in locations where SEZs expand access to local markets and where there are more dynamic labor environments. The SEZs also lead the microfinance lender to improve credit terms, which contribute notably to improved enterprise outcomes. To broaden the relevance of our findings, we demonstrate how related sub-national business environment factors help explain different borrower outcomes found in extant studies that measure microfinance impacts. Policy implications are that an important subset of microfinance borrowers is able to seize opportunities provided by local business environments, and that national and subnational governments can play active roles in facilitating such improved business environments for micro and small enterprises.

## Introduction

Growth of micro- and small enterprises (MSEs), improved productivity and income generation, and job creation are expected consequences of improved financial access for unbanked or underbanked populations, according to the financial inclusion literature’s traditional theory of change (Armendáriz & Morduch (2010)). This theory has been subject to vigorous debate. On the one hand, a number of prominent randomized controlled trials (RCTs) on microcredit have shown muted average effects on entrepreneurial and poverty outcomes, albeit they find notable heterogeneity across and within studies (Banerjee et al. (2015b)). On the other hand, there is more positive evidence on real economic outcomes provided by studies on large-scale microfinance or rural bank expansion (Burgess & Pande (2005); Bruhn & Love (2014)). The mixed nature of this evidence has led scholars to comment that capital constraints may not always be the primary explanation for the weak outcomes for many MSEs and to suggest that greater emphasis be placed on understanding the drivers of differential outcomes (Banerjee et al. (2015a); Pritchett & Sandefur (2015); Morduch (2020)).

In particular, A. Banerjee (2013) suggests that a key constraint for many microfinance clients may instead be factors related to the quality of the business environments in which they operate. Despite the intuitive nature of the suggestion and its relevance to microfinance’s theory of change, the empirical literature on microcredit to date still largely overlooks this. This is perhaps due to difficulties in experimenting on broader business environment factors—typically beyond researchers’ control—or being able to capture sufficient variation and a meaningful counterfactual group if drawing on observational data (as mentioned by Deaton & Cartwright (2018) and Meager (2019)). By contrast, the enterprise growth literature has given much more attention to the role of such environmental factors (see Wiklund et al. (2009) for a literature overview) and provides a useful foundation for examining this research angle. Nevertheless, the evidence base it provides still disproportionately focuses on formal and larger enterprises and those located in developed or emerging economy settings, rather than MSEs in lower income economy settings, which form the core clientele of microfinance institutions.

Our paper consequently seeks to fill an evidence and research gap by answering the following main research question: *How did the implementation of Cambodia’s Special Economic Zones impact enterprise growth outcomes of MSE microfinance clients?* As elsewhere, Cambodia’s special economic zones (henceforth, SEZs) are subject to different laws and regulations from those pertaining to other areas of the country, which we posit have led to improved business environments in the form of agglomeration effects and institutional “framework conditions.” For example, SEZs are commonly considered to provide a supply-side shock by improving access to various business inputs. This includes improved capital availability from both investors driven by tax and fiscal incentives, and from lenders driven by expectations of improved business opportunities.^1^ SEZs are also expected to produce a demand-side shock by improving access to external markets for enterprises’ goods and services. For example, this could be due to improvements in transportation networks or better cross-border collaboration (e.g., reduced bureaucratic red-tape for customs, trade, and foreign exchange). In the case of Cambodia, this is expected both for country-border SEZs, which have gained increased linkages in South-east Asia via development of the Greater Mekong Economic Sub-corridor, and for interior and ocean border SEZs, which are heavily orientated toward global garment exports and have benefited from development of investment in deep-sea ports (Abonyi et al. (2016)).

To study this in practice, we draw on a research partnership with a leading Cambodian microfinance provider and the staggered roll-out of Cambodia’s SEZs between 2011 and 2016.

We use a proprietary data sample of entrepreneurial clients that is broadly representative of the provider’s micro- and small enterprise clients. Second, we combine this with data on SEZs from Open Development Cambodia and contextual information on Cambodia’s administrative areas from the Cambodian General Population Survey. Using this merged data, we map our research partner’s branches and client data to SEZ locations at the district level. To mitigate concerns that ex ante contextual characteristics of SEZ locations may differ and drive differential MSE client outcomes, we apply propensity score weighting to construct a synthetic counterfactual group based on contextual characteristics known to drive SEZ placement. We combine “inverse probability of treatment weighting” (IPTW) and generalized difference-in-differences (DiD) methods for our main analysis. We then set up additional empirical tests to explore mechanisms explaining the different business growth outcomes for exposed versus unexposed MSE microfinance borrowers, looking at client-level, environment-level, and credit provision factors.

Our key findings are severalfold. First, our descriptive evidence on enterprise growth in both absolute and relative terms shows that MSEs at both ends of the distribution—roughly the top and bottom 10 to 15 %—exhibit considerable growth and decline, respectively. Specifically, our absolute measure captures the raw change in number of employees between borrowers’ consecutive loan cycles, whereas our relative measure applies a logarithmic transformation of the number of employees followed by first differencing of the log values (which is roughly equivalent to percentage differences). This is in contrast to the fairly small mean employment growth that we find for either the treatment or control MSEs. On the one hand, this fits with most microfinance studies in suggesting that the majority of microfinance clients may not necessarily be motivated toward or capable of growth, and that the likelihood of transformative changes is fairly small *for the average client*. On the other hand, our finding is well in line with stylized facts from the enterprise growth literature. While this further motivates a need for changes to microfinance’s traditional theory of change (which has already been evolving to capture heterogeneous client motivations and types), it also suggests that entrepreneurial microfinance borrowers should not be summarily considered less capable of growing their businesses than firms in other settings.

Second, our main empirical result is causal evidence that SEZ implementation had a statistically and economically significant positive impact on employment growth among MSE microfinance clients. In our setting, we estimate that the positive shock from SEZ implementation increased employment generated by micro- and small enterprises in relative annualized terms by roughly 15 % for MSEs in SEZ districts, in comparison with MSEs in control districts. In absolute terms, an interpretation of the economic magnitude of the effects on MSEs—which considers the average initial starting size of businesses with 1.7 employees—is that every 10 micro and small businesses in the SEZ districts started out with approximately 17 employees *combined* and ended up with between three to five additional employees *combined* compared to similar businesses in the control districts, following SEZ implementation.^2^ The main results are quite robust to model specifications and a variety of sensitivity checks.

Next, we set up additional tests on enterprise-, district-, and loan-level factors that led to differential effects to better understand the mechanisms driving the improved growth outcomes. At the client level, we observe that growth driven by the onset of SEZs is particularly pronounced for agricultural (and to a lesser degree service) enterprises, particularly when sub-setting the analyses to districts bordering with Thailand or Vietnam. This is in line with theory and anecdotal evidence that those particular SEZs generated a demand-side shock, so that Cambodian enterprises were able to leverage their cheaper production costs to gain new markets for agricultural goods in their more developed neighbors. We also observe some signs that women-owned enterprises saw significantly lower growth outcomes following SEZ implementation, which is consistent with them facing additional barriers to economic and social participation (as has been previously documented in the Cambodian setting). At the district level, our findings support the intuition that the size of local economic markets with more dynamic labor pools and stronger connections to markets matter for providing growth opportunities for local businesses, particularly when combined with SEZs. Our findings also suggest that SEZ implementation led to a supply-side shock where local branches of our partner micro-finance provider notably improved key loan terms for borrowers, without necessarily seeing changes in credit demand or composition of incoming borrowers. Borrowers who received improved loan terms exhibited significant greater business growth in terms of employment.

Finally, we explore how our findings help explain varied business outcomes exhibited by sample borrowers from a number of widely cited microcredit studies. We combine their data and construct additional indicators on business environment factors related to the expected causal mechanisms underlying our hypotheses. These include subnational-level proxies for the following: (1) levels of financial and human capital; (2) quality of local institutions and rule of law; (3) access to better infrastructure; (4) general business environment indicators; and (5) market size. This secondary analysis confirms the importance of a number of the aforementioned framework conditions in predicting differential client business outcomes in these past studies, beyond employment indicators. For example, we find that microcredit clients in areas with higher ex ante per capita outstanding debt exhibit significantly higher ex post growth in terms of both employment and profits. Moreover, areas with higher ex ante trust in formal institutions are particularly strongly related to greater levels of employment in client businesses, whereas areas with access to better infrastructure and larger markets show signs of particularly higher client profits, respectively. The combined findings underscore a general point that transformative entrepreneurial outcomes for the clientele of microfinance institutions are more likely to occur when broader business environment conditions are also favorable.

Our paper relates to several streams of literature. First, it contributes to the large body of literature on special economic zones. While this literature has generated a wide body of theory and descriptive evidence (see, for example, Johansson & Nilsson (1997a); Akinci & Crittle (2008); Baissac (2011); Moberg (2015)), it has only recently begun to assess dynamic impacts of the zones on local firms using rigorous empirical methods. Within the empirical studies on dynamic effects from SEZs, our paper is methodologically closest to Steenbergen & Javorcik (2017), who, like us, focus on analyzing firm-level impacts, and combine propensity score matching (PSM) and difference-in-differences to improve identification. However, their data sample consists of export-oriented medium-sized enterprises (generally, more than 50 employees). To the best of our knowledge, ours is the first study that attempts to estimate dynamic effects of SEZs on local micro- and small enterprise-level outcomes in a developing country context.^3^ Ours is also one of the few studies in this literature, apart from Frick et al. (2018), to empirically study how different SEZ and territorial factors may influence the intensity of dynamic effects, where we again differ in using enterprise-level outcomes. This allows us to go further than most of the existing studies in demonstrating some of the key mechanisms at play that help drive the improved business outcomes.

Secondly, our paper contributes to the literature on financial access, in which it is the first empirical study—to our knowledge—that focuses explicitly on the role of contextual aspects of business environments in explaining the entrepreneurial outcomes of microfinance clients. The study that perhaps has the closest intuition is Ashraf et al. (2009), who focus on the role of improved contextual opportunities through improved value-chain linkages to new markets. In this literature strand, there are also a number of studies analyzing the effects of large-scale bank expansion on financial inclusion and development (Burgess & Pande (2005); Bruhn & Love (2014)). In contrast with microfinance RCTs, these large-scale expansion studies have documented more sizeable economic benefits for previously unbanked populations. Our results bridge between these two literature substrands. Like the RCTs, we find that the average MSE’s business exhibits small change and that the majority of growth occurs for a small but important subset of enterprises. However, we also observe that at a nationwide scale, MSEs in more opportune business contexts are more readily able to exhibit transformative growth and create employment for others. Real outcomes driven by this narrower subset of MSEs and locations would presumably be less likely to be picked up in RCTs, which tend to be more localized, but easier to detect in studies that are done at nationwide scale and/or which factor in general equilibrium effects (e.g., Breza & Kinnan (2021)).

Finally, the study also contributes more generally to a rich strand of enterprise growth literature that focuses on the role that environmental factors play in explaining business outcomes. This literature has assessed how different environmental aspects, such as firm location (Giner et al. (2016)), industry (Coad (2009)), markets and agglomerations (Stam (2010); Audretsch et al. (2012)), and institutional factors (Acemoglu et al. (2014); Krasniqi & Desai (2016)), affect both the growth orientations and actual growth of businesses. However, it has had a disproportionate focus on formal and larger enterprises and those located in developed or emerging economy contexts. One of the reasons for this, apart from greater difficulty with data access, may be that micro-enterprises and developing economies, which form the bulk of the target clientele of microfinance institutions, are generally expected to be less conducive to growth (Bourlès & Cozarenco (2018)). Irrespective of the reason, this may limit transferability of findings from these prior studies. To this end, our study provides evidence on the relative share of growth-orientated microfinance clients from one of the largest microfinance markets globally. Moreover, we give evidence on some of the specific business environment conditions which help improve their likelihood of growth.

The remainder of the paper proceeds as follows. Section 2 reviews the relevant literature and outlines the hypotheses we seek to test. Section 3 describes our empirical setting and Section 4 the study’s data sources and the construction of treatment and control groups. Section 5 presents the paper’s main empirical analysis and results. Finally, Section 6 discusses the broader relevance of our results in relation to prior microcredit impact evaluations and makes concluding remarks.

## Literature and hypotheses

This paper draws on several literature streams to develop our hypotheses. First, we draw on the literature on entrepreneurial outcomes from access to finance. Second, we build on a large body of literature on business environments from the enterprise growth and institutional economic literature, which also includes a distinct strand of literature on special economic zones.

### Access to financial services and entrepreneurial outcomes

To study MSE growth, we draw from the literature on the impact of access to finance. This discourse was particularly influenced by results from a seminal group of microcredit RCTs (see Banerjee et al. (2015b)). Based on a comprehensive review of the broader literature on the impact of microcredit, Beck (2015, p. 20) states that “[a] conclusion would be that effects are typically statistically and economically more significant for individual or household level outcomes than on the microenterprise level.”

More recent contributions, however, highlight that outcomes for entrepreneurial clients are quite heterogeneous both within studies (Banerjee et al. (2015a); A. Banerjee et al. (2019)) and across studies (Pritchett & Sandefur (2015)). First, as noted by Banerjee et al. (2015b) and Pritchett & Sandefur (2015), RCTs’ results and evaluations focus on average treatment effects, which obscures evidence that some clients in certain studies do exhibit transformative business outcomes. For example, Banerjee et al. (2015a) undertake the longest-term study, considering the effect of a randomized MFI branch expansion in Hyderabad, India, and find that 3 years in business growth in the treatment areas are larger, but the effect is concentrated among a subset of highly successful clients with pre-existing businesses. Second, across the studies, some find evidence that is in line with the original idea of microcredit supporting or improving existing entrepreneurship (e.g., Augsburg et al. (2015); Attanasio et al. (2015); Angelucci et al. (2015)), whereas others do not (e.g., Tarozzi et al. (2015); Karlan & Zinman (2011)).

To better understand the reasons for differential effects, including individual-, business-, or loan-level factors, a number of studies have coupled loan products with interventions to mitigate low financial literacy or poor business management skills, to mixed results. For example, Karlan et al. (2015) and Karlan & Valdivia (2011) conduct RCTs in Ghana and Peru, combining access to finance with consulting or entrepreneurial training, but find no evidence of improved business revenue, profits, or employment. De Mel et al. (2014) find that combined business training and grant money for a group of female entrepreneurs increase short-term profitability for existing entrepreneurs and longer-term profitability for first-time entrepreneurs. Meanwhile, A. Banerjee (2013) points out that while many archetypal microfinance lending practices (such as starting with small loan sizes, short maturity, frequent and fixed repayment schedules, joint liability) may be useful for mitigating information asymmetries, they may also be explanations for why these loans have not generated more impact for borrowers.

Broader environmental features faced by microcredit clientele remain understudied; nevertheless, they may intuitively play important roles in constraining individuals’ opportunities and motivations for business growth. RCTs tend to be fairly localized and are typically limited to studying shorter-term (1-to-2 year) impacts of single interventions (Armendáriz & Morduch (2010)) or rely on identifying narrow subsets of clients on the margins (Augsburg et al. (2015)).^4^ This arguably makes them less useful for evaluating how broader factors, such as the local business environment context, may help explain the outcomes of microcredit borrowers, even though A. Banerjee (2013) proposes that these factors may actually pose greater constraints. We therefore start by postulating a proposition that guides this paper’s analysis:

- *Proposition*: *Microfinance MSE client growth is conditional on opportunities provided by local business environments.*

### Local business environment and special economic zones

The empirical literature on enterprise growth has studied factors related to local business environments more closely. This includes a large body of literature that examines the roles of territorial contexts and institutional quality, as well as a subset that focuses specifically on special economic zones.

Scholars that focus on territorial aspects document that higher-growth firms are disproportionately concentrated in urban areas and areas with a higher population density (see, for example, those described by Liedholm (2002); Acs & Mueller (2008); Coad (2009); Clarke et al. (2016)). Stam (2010) highlights various advantages stemming from higher population density areas, including the relative ease of access to markets and customers as well as to the inputs required to produce goods or services. Moreover, some scholars have focused on the role of agglomerations—for example, industrial districts, local manufacturing systems of large firms, and technological districts—which are expected to lead to “agglomeration effects,” including improved integration into larger and external markets, reduced costs of input, and productivity spillovers (Porter (1998); Audretsch et al. (2012); Giner et al. (2016)). In parallel, new institutional economics literature has long emphasized that it is local institutions which ultimately dictate whether entrepreneurial activity is allocated to productive, unproductive, or destructive purposes within a given economy (North (1991); Acemoglu et al. (2014)). The implications for policymakers are that establishing conducive framework conditions can be a more effective way of preparing fertile ground upon which winners may pick themselves. A growing body of evidence documents that the quality of the business environment—institutional factors such as governance (including efficiency of government administration and the rule of law), infrastructure, and access to markets—can notably differ at subnational levels and have an important influence on the nature of entrepreneurial activity (Hallward-Driemeier et al. (2006); Malesky (2009); Bjørnskov & Foss (2016); Krasniqi & Desai (2016)).

A related but distinct body of literature has developed around special economic zones, which are explicitly designed to directly improve many of these territorial and institutional framework conditions.^5^ For example, SEZs are usually created in order to facilitate rapid economic growth in certain geographic regions. In practice, they can use a range of methods to improve access to supply-side inputs for business, such as fiscal incentives to attract greater capital and technological inflows, and developing local infrastructure (e.g., electricity, water, ICT). Moreover, they can improve demand-side access to markets by setting up cross-border collaboration, external transportation networks, and bureaucratic efficiency through one-shop administration. Theoretical literature also highlights that SEZs may create further dynamic effects within the surrounding domestic areas via linkage and spillover effects (Baissac (2011)). “Linkage effects” refer to smaller domestic firms, both informal and formal, gaining increased opportunities to integrate into larger markets. SEZs can help drive productivity upgrading—for example, through transfers of human capital and technology. Meanwhile, indirect “spillover effects” refer to zones coming to rely upon and help develop their surrounding area with respect to basic needs for accommodation, consumer services, and other place-specific, non-zone activities.

Several recent empirical studies provide some causal evidence of dynamic effects, albeit they thus far generally provide stronger evidence of linkage effects compared to spillover effects. Wang (2013) uses a difference-in-differences approach to demonstrate positive dynamic impacts of Chinese SEZs on neighboring municipalities, as measured by domestic investment, TFP growth, and local factor prices. Ciz˙kowicz et al. (2016) apply fixed effects models to a firm panel dataset from Poland and find that SEZs have substantial positive effects on employment, for both the SEZ firms and non-SEZ firms in the host counties. Finally, Alkon (2018) combines a subdistrict-level dataset on Indian SEZs and census data and creates a synthetic control group using propensity score matching. In contrast to the previously cited authors, he finds that the Indian SEZs have generally created insignificant spillover effects on local socioeconomic development, as proxied by a variety of district-level social and economic developmental variables, such as education and transport infrastructure, and percentage of marginalized households.

### Hypotheses

As suggested by the previous discussion, enterprise growth has often been studied in a fragmented way, with different theoretical perspectives highlighting distinct factors as playing a primary and independent role in explaining or constraining growth. For example, common factors that receive attention in related literature include entrepreneurial orientation and growth attitudes, resource constraints (i.e., in particular, financial, human capital, or networks), and broader environments. However, there are also some scholars that combine these fragmented perspectives into *integrative frameworks*, noting that these perspectives are not necessarily independent of each other and that they may lead to conflicting hypotheses about growth under certain combined circumstances (see, for example, Wiklund et al. (2009); Nichter & Goldmark (2009); Davidsson et al. (2010)). In such views, the focus is on understanding the interdependencies between factors that help explain differential growth.^6^

Our paper is in line with this perspective of *integrative frameworks* and draws on unique aspects of our study setting to better understand the nexus between access to finance and business environments on entrepreneurial growth outcomes. In our study, we take as our starting point MSEs who have all gained access to formal financial services and are at face value less immediately constrained by financial resource limitations. Albeit, as we will later expand upon, they differ notably in terms of the quality of their financial access. These MSEs are exposed to policy-induced improvements to their surrounding local business environments, which is catalyzed by SEZ implementation. Like many developing and emerging economy settings, development in Cambodia remains uneven across regions and households, with a weak overall business environment cited as a leading barrier to greater growth and opportunities (Malesky (2009). For example, rural and informal enterprises in Cambodia have been found to be disproportionately affected by weak public service delivery (institutions), as well as limited access to organized markets and poor infrastructure (i.e., territorial factors); these are thought to be key constraints on their opportunities for growth (Asian Development Bank (2016)). Given that SEZs are directly aimed at mitigating these types of barriers, we start by testing our main hypothesis:

- *H*_1_: *The implementation of SEZs as a means to improve the business environment has positive effects on the business growth of microcredit clients in these zones.*

#### Individual or business-level factors combining with SEZ implementation to drive differential growth

We develop a number of additional hypotheses that help us better understand how the SEZ implementation may lead to improved enterprise growth. First, we develop secondary hypotheses related to individual or business-level factors that may interact with SEZ implementation. Following the enterprise growth literature (Ayyagari et al. (2011)), different types of MSEs and individuals may experience greater benefit from SEZ implementation based on some aspects of their starting context (e.g., whether they started off initially larger, whether they are in the right economic sector to capitalize from the SEZ, whether they are run by population segments that face additional constraints).

We follow the suggestion of Beck (2015), who argues that small rather than micro enterprises might have more potential to be transformative and create jobs. This is in line with theoretical literature in which a dual-economy model exists where larger firms are initially subject to constraints and regulations (e.g., inefficient bureaucracy, corruption) that smaller firms are able to evade, but such constraints can be subsequently relaxed or mitigated (Harris & Todaro (1970); Krueger (2013)).^7^ In the Cambodian setting, the larger majority of firms (roughly 90%) are micro enterprises (Malesky (2009)). While Cambodia’s micro- and small enterprises share some key constraints, studies have documented that micro-enterprises are more likely to be hindered by limited entrepreneurial capacity (Ayyagari et al. (2011); Gherhes et al. (2016); DESA (2022)), whereas small and medium enterprises are particularly affected by uncertainty in the business climate. In Cambodia, this is found to be due to a combination of poor governance, corruption, weak courts, and incomplete infrastructure (Baily (2007); Malesky (2009)). In sum, SEZs more directly target these primary constraints of small enterprises, and we posit that they are more likely to benefit from SEZ implementation.

- *H*_2_*a*: *There is a positive differential in business growth outcomes from SEZ implementation for small enterprise clients compared to microenterprise clients.*

Next, as noted earlier, the SEZ literature has provided growing empirical evidence that they can improve outcomes for firms centered around SEZ-related economic activities (Wang (2013); Ciz˙kowicz et al. (2016); Steenbergen & Javorcik (2017)). However, this literature has thus far found less substantial evidence of indirect spillover effects. In Cambodia, the SEZs have been heavily orientated around developing regional linkages in Southeast Asia for agricultural firms and global linkages for heavier manufacturing (particularly garments) firms, for example, through improved cross-border infrastructure and bureaucratic links with neighboring countries (Sisovanna (2012); Warr & Menon (2016)). We thus posit that borrowers whose businesses fall in the agricultural and manufacturing sector (as well as those who trade those goods) will likely exhibit greater growth driven by SEZ implementation than those whose businesses are in the services sector. This is in line with Khandelwal et al. (2018), who suggest that one of the main benefits of SEZs is that they facilitate knowledge transfers, between foreign companies in the SEZ and local companies (and local staff) in the same sectors of activity.

- *H*_2_*b*: *There is a positive differential in business growth outcomes from SEZ implementation for production sector clients (agriculture, trade, and manufacturing) compared to service sector clients.*

Finally, like in many settings, women in Cambodia have historically faced barriers to equal economic, social, and political participation. For example, prior studies in Cambodia have documented that long-standing culturally defined behavior norms for women (known as the *Chba’p*) create gender inequalities in education and occupational choice. This has wide-ranging repercussions for women including reduced opportunities for higher paying and managerial/leadership positions, heavier (unpaid) household work burden, and less access to necessary resources, such as finance (UNIFEM (2004); Asian Development Bank (2015)). This creates additional constraints for women entrepreneurs to succeed in their businesses. On the one hand, some recent studies on Cambodian SEZs have found evidence that they have helped improve women’s economic empowerment, albeit this is explained primarily by increased female labor force participation rate and wages (as employees of SEZ-based firms) (Brussevich (2020)). On the other hand, these studies do not investigate or capture any differences in impacts on business owners (employers) across gender. In practice, Cambodia’s SEZ policies do not appear to target any of the underlying structural reasons hindering women’s entrepreneurial opportunities, at least in the short term. For this reason, our *a priori* expectation is that male-owned enterprise borrowers will be relatively better positioned to benefit from SEZ implementation:

- *H*_2_*c*: *There is a negative differential in business growth outcomes from SEZ implementation for women clients compared to male clients.*

#### District or SEZ-level factors combining with SEZ implementation to drive differential growth

The geographic coverage of our sample captures considerable variation in terms of the type of territorial context (e.g., market size and type of location) and in terms of aspects of how SEZs were implemented (e.g., the timing of SEZ starts relative to our study period, distance to the individuals’ locations). We leverage this variation to similarly develop secondary hypotheses related to district or SEZ-level factors that may drive differential growth outcomes.

First, we test a stylized fact that higher-growth firms are disproportionately concentrated in areas with higher population density and growth (which has been found in a large body of studies—e.g., Liedholm (2002); Acs & Mueller (2008); Coad (2009)). As highlighted by Stam (2010), the mechanisms explaining this could be improved access to markets and customers as well as to the inputs required (for example, capital, skilled labor/employees, suppliers) to produce goods or services. There is recent empirical evidence supporting the importance of these factors in the context of SEZs from Frick et al. (2018), who test a range of agglomeration-related SEZ factors (including access to markets, human capital, and population density, SEZ size, and services offered) on a global dataset of SEZs and nightlight data, and find strongest effects from proximity to larger cities or markets.

- *H*_3_*a*: *There is a positive differential for MSE microcredit clients in areas with higher population density and population growth.*

Second, we note that in the context of Cambodia, a portion of SEZs have been specifically set up to leverage cross-border trade and cooperation with Cambodia’s neighbors, Thailand and Vietnam. We examine whether there are differential growth outcomes in such settings, given that the respective countries have been working steadily toward improving cross-border cooperation (Abonyi et al. (2016)) and that there has been anecdotal evidence of notable increases in cross-border trade in agricultural and other goods.^8^ We expect that firms in such SEZ locations may subsequently be better able to leverage agglomeration and network effects stemming from increased access to cross-border inputs and external markets.

- *H*_3_*b*: *There is a positive differential for MSE microcredit clients in areas located in a country border area compared to an interior area.*

We also develop hypotheses on SEZ implementation characteristics, namely their maturity and proximity to clients. The first of these hypotheses follows the findings of Farole (2011), who do a cross-country and long-term comparison of SEZs, and finds that many successful SEZs have required an incubation period, with slower growth in the initial years that then grows exponentially once significant time passes—i.e., as SEZs become increasingly “mature.” This is also in line with stylized facts from the enterprise growth literature, where many studies find employment growth to be sporadic and often involving one-off expansion more readily observed over longer time scales (Coad (2009)).

- *H*_3_*c*: *There is a positive differential in business outcomes for MSE microcredit clients as time from SEZ creation increases.*

The last hypothesis is developed under the more general agglomeration-related reasoning that benefits to the local economy from the SEZ will be more likely to occur in closer proximity to the zone itself. That is, we want to test that the further one moves from the zones, the weaker any positive impact of the SEZs on local firms will become.

- *H*_3_*d*: *SEZ effects are stronger when in closer proximity to MSE microcredit clients.*

#### Loan-level factors combining with SEZ implementation to drive differential growth

Finally, our sample individuals all ostensibly have access to credit but in practice differ considerably in their quality of access (i.e., in terms of their relationships with lenders and the loan terms that they receive). We leverage this variation to develop several secondary hypotheses related to loan-level factors that may drive differential growth outcomes. This is relevant to study in closer detail as Banerjee et al. (2015a) find evidence of heterogeneous (and larger effects) for intensive margin microcredit borrowers. The banking literature has also established how length and strength of borrower-lender relationships can be linked with a variety of improved firm outcomes, where the theoretical mechanisms include both improved terms of finance (Kysucky & Norden (2016); Delis et al. (2017)) and provision of “soft guidance” to enterprises (Brancati (2015); López-Espinosa et al. (2017); R. N. Banerjee et al. (2021)). We posit that clients with longer relationships with the lender will be better positioned to seize growth opportunities after SEZ implementation.

- *H*_4_*a*: *There is a positive differential for intensive margin compared to extensive margin clients following SEZ implementation.*

In a related sense, A. Banerjee (2013) has posited that many archetypal microfinance lending practices (such as starting with small loan sizes, short maturity, imposition of joint liability or more severe collateral) may restrict impact for borrowers. A number of experimental studies on archetypal microfinance lending technologies focus on demonstrating how relaxing some of the rigid microcredit terms and conditions can lead to improved borrower outcomes, by being better suited for capitalizing on high-growth opportunities (see, for example, Field et al. (2013) and Giné & Karlan (2014)). We draw on our unique institutional setting to analyze the impact of combining both improved loan terms and environmental conditions on local entrepreneurs.

- *H*_4_*b*: *There is a positive differential on business outcomes for MSE microcredit clients who receive better credit terms and conditions following SEZ implementation.*

## Cambodia’s special economic zones

Cambodia’s SEZs provide a useful policy shock to test whether local MSEs have responded to various improvements in the local business environment. Cambodia’s government set up a special legal form in 2005 allowing private operators to establish SEZs to help address uneven regional growth and development (Abonyi et al. (2016)). From 2005 to 2016, 34 SEZs were set up in different districts and years (Open Development Cambodia (2017)) (see Appendix Table 12 for a full list). As elsewhere, Cambodia’s SEZs are subject to different laws and regulations from those pertaining to other areas of the country, and these are expected to improve both supply-side access to inputs and demand-side access to markets for the goods and services being produced by local firms (Warr & Menon (2016)).

On the supply side, the SEZs allow fiscal incentives to investors and firms, including guarantees of no price or foreign exchange controls, free remittance of foreign currency, exemption from import duty and value-added tax (VAT), a 20% corporate tax rate, and tax holidays of 9 years for the zone developer and of variable length for other firms located inside the zone. This is expected to channel capital from domestic and foreign investors and local lenders and improve access to necessary business inputs and technology. SEZs are also required to set up local infrastructure to meet minimum standards—that is, local road networks, electricity, sewage treatment, water, and telecommunications, all of which have been found in other studies in Cambodia to be important supply-side constraints for local firms (see, for example, Malesky (2006, 2009)).

On the demand side, the SEZs are expected to improve access to external markets for local firms’ goods and services. For example, Cambodia’s SEZs have been set up to simplify customs and other bureaucratic processes via specially trained government officials on-site to provide a “one-stop shop” for all administrative services. This is particularly the case with the SEZs along the border with Thailand and Vietnam, where the respective governments have set up policy initiatives for regional integration to promote economic growth, economies of scale, and contribute to poverty reduction (Sisovanna (2012)). Furthermore, Abonyi et al. (2016) note that the SEZs have helped contribute to development of the Greater Mekong Subregion (GMS) economic corridors and deep-sea ports, which have made it easier for local firms to serve regional markets in Southeast Asia and globally.

Generally, the Cambodian SEZs are in line with a current broader conception of SEZs, which attempts to move beyond more narrowly framed export processing zones (Johansson & Nilsson (1997b)) and be envisioned to holistically develop areas for services and residence as well (see Akinci & Crittle (2008) on the evolution and typology of SEZs). Cambodia’s SEZs differ from other regional SEZs in Southeast Asia and those studied in the empirical literature. Abonyi et al. (2016) compare Cambodia’s SEZs to other local SEZs in Southeast Asia, which collectively create the GMS economic corridor. They note that the activities in SEZs in Lao PDR have up until recently been restricted to hotel and casino complexes and those in Myanmar have largely been unable to generate sufficient investment and interest due to political unrest. Meanwhile, the SEZs in Thailand and Vietnam have considerable similarities in orientation and regulation but are dissimilar as they are much larger in area and generally focused on slightly higher value-added activities. Moreover, the SEZs studied in the Chinese context by Wang (2013) and Indian context by Alkon (2018) are more in line with narrowly framed traditional export processing zones. The Cambodian SEZs are thus most similar to those studied in the Polish context by Ciz˙kowicz et al. (2016) and in the Rwandan context by Steenbergen & Javorcik (2017), as they are broadly envisioned, accommodate all types of activities, and have a much broader set of incentives and benefits.

Prior case studies have found that Cambodia’s SEZs have been modestly successful in providing high-quality waged employment and attracting foreign investment (Open Development Cambodia (2017)). Moreover, they play useful roles as enclaves that provide a stable business environment, reasonable infrastructure, and less red tape (Abonyi et al. (2016)). Warr & Menon (2016) estimate around 70,000 waged jobs created in SEZ-located firms up to and including the year 2015.^9^

## Data

In this section, we briefly describe the data sources used for the study’s analysis, elaborate on the establishment of treatment and control groups, and provide descriptive statistics on client- and territorial-level characteristics.

### Data on Cambodian enterprises

To obtain data on entrepreneurial clients, we partner with a leading Cambodian financial service provider and extract a stratified random sample of business loan client records from its management information system (MIS). Our research partner is one of the market leaders in terms of clients served and volumes of savings and loans, has nearly nationwide outreach with branches in the majority of Cambodia’s 25 provinces and districts, and has a diverse lending portfolio covering micro- through medium-sized enterprises. Thus, our sample is less likely to be biased by attrition of growth entrepreneurs switching to suppliers of larger loans. The MIS’s various modules capture key information on clients, business, and loan characteristics. This information is updated by loan officers and recorded at the initiation of every loan cycle for a client, allowing changes in key business growth indicators and other characteristics to be tracked across time. The sample is broadly representative of the provider’s micro- and small enterprise clientele in the period January 2011 to December 2016.^10^ Appendix Section D provides details of the sample design and methodology of extraction.

We use data from the business module to construct the paper’s main dependent variable, which is growth in the number of employees between loan cycles.^11^ Drawing on lessons from the empirical enterprise growth literature, we measure this in terms of both absolute and the relative (logarithmic) change.^12^ Specifically, our absolute measure captures the raw change in number of employees between the consecutive loan cycles, whereas our relative measure applies a logarithmic transformation of the number of employees followed by first differencing of the log values (which is roughly equivalent to percentage differences). Since the dataset is an unbalanced panel (and there may be different lengths of time between consecutive loan observations), the time unit of the growth measure is normalized into an annual growth rate by dividing the relative change between consecutive loans by the amount of time between the application dates of the loan cycles. Furthermore, to reduce the chance that annualized rates may exaggerate growth if a client received two consecutive loans within a short time period, we apply the additional criterion that a minimum of 12 months must pass between consecutive loans for the associated growth observation to be included in the data sample. As summarized by Davidsson et al. (2010), methodological choices related to these factors have been shown to have an important influence on results. Thus, we conduct a number of sensitivity checks in Appendix 3 to analyze how each separate methodological issue affects results.^13^

The data also allow us to control for or explore the role of a variety of client-, business-, and loan-level characteristics drawn from the relevant literature that are cited as leading to differential effects on client outcomes. At the client level, this includes gender (McKenzie et al. (2008); Banerjee et al. (2015a)), age (Fajnzylber et al. (2006)), whether they are an individual household or have dependents, and whether they have a single business activity or are simultaneously managing multiple activities. At the business level, this includes whether the business is a micro- or small enterprise (Mead & Liedholm (1998)) and the economic sector of the business (Coad (2009)). Finally, at the loan level, we use the loan cycle number as proxy for the length or strength of the borrowing relationship and key loan terms, such as loan amount, interest rate, loan duration, and percent of loan amount collateralized. Further details on variable construction are in Appendix D.3.

### Data on Cambodia’s special economic zones and districts

For data on special economic zones, we draw on an SEZ database maintained by Open Development Cambodia^14^ that provides an up-to-date list of SEZs with their respective start dates and addresses, among other information. We use this to map SEZ locations to our research partner’s branches at the district level and construct a dummy variable at the district and year level for the date of a given SEZ’s implementation. This dummy variable, which indicates the interaction between treatment group and post-treatment period, is used as the main explanatory variable in this paper’s empirical analysis.

Meanwhile, for data on district characteristics, we draw on data from the Cambodian General Population Census, which was conducted by Cambodia’s National Institute of Statistics in 1998 and 2008 (with a partial intercensal census in 2013).^15^ We use this census data to compile information on population size, land area, population density, and population growth. We similarly map these data at the district level to the SEZs and our research partner’s branches to analyze the contextual background of districts that are included in our study sample.

We choose to use districts as the territorial level for separating branch clients into treatment and control groups for the paper’s analysis because this mirrors the branch structure of our research partner, which typically has one branch office per district.^16^ The few exceptions where there is more than one branch per district are in the three main urban agglomerations in Cambodia—Phnom Penh, Siem Reap, and Battambang, where the population density is high enough in a few instances to support such increased density of branches within a single district.

Of our research partner’s 126 branches in the full data sample, 20 are located in districts where an SEZ has been implemented. Among these, nine branches are in locations where the SEZ was implemented during our study period (January 1, 2011, to December 31, 2016) and the SEZ is the first ever to be implemented in the district. It is worth specifying that we use all 20 branches and districts where SEZs were implemented for estimating propensity scores for SEZ implementation since it helps improve the precision of the model. However, to avoid the possibility of confounding effects on business outcomes from previous SEZs, clients from branches located in districts with SEZs implemented before 2011 are omitted in the later difference-in-differences analysis on employment growth.^17^ A list of Cambodian SEZs and details of which are included in our main analysis are provided in Appendix Table 12. A key takeaway is that there is both geographical and temporal variation in the implementation of SEZs during the study period, which our main analysis exploits for its empirical strategy.

### Establishing treatment and control groups

The study’s treatment group consists of micro- and small business loan clients from nine of our research partner’s branches in districts where an SEZ was implemented for the first time between 2011 and 2016. As described in Section 2.2, the placement of SEZs—both in Cambodia and more generally—tends to follow some established patterns (that is, it is non-random). This may lead to concerns that different ex ante contextual characteristics of the SEZ districts bias estimates from the paper’s main analysis. To better mitigate these concerns, we use inverse propensity for treatment weighting to create a synthetic control group. This control group mimics key contextual factors that are expected to influence the likelihood of SEZ presence, based on the literature, government records, and demonstrated through exploratory analyses of our data. For example, Abonyi et al. (2016) and Warr & Menon (2016) highlight that Cambodian SEZ locations must meet minimum land area requirements (of more than 50 hectares) and have been typically selected based on balancing proximity to major transportation infrastructure (whether roads, ports or potential port locations, or airports), availability of labor, and access to secondary suppliers (i.e., other manufacturers and services).

Drawing on these sources on the placement of Cambodian SEZs, we first run a probit regression at the district level to obtain propensity scores for SEZ placement, based on a district’s land area, population density, and country region, and dummy indicators for whether a district is an urban location, situated on country or ocean borders, or along the Greater Mekong Sub-region economic corridor. Table 1 summarizes results from this propensity score model and demonstrates that the combined regressors are jointly highly predictive of SEZ placement. For example, the *χ*^2^ value confirms that we can reject a null hypothesis that the included variables do not jointly explain SEZ placement. Meanwhile, the pseudo-*R*^2^ indicates that the model regressors explain roughly 30 % of the probability of SEZ implementation.

**Table 1:** Propensity score model estimating predicted probability of SEZ placement based on district-level features. This table presents coefficient estimates for a probit model estimating the predicted probability of SEZ placement based on district-level contextual features. The full sample includes 126 branches-districts, of which 20 are SEZ district locations

Probit Regression				# of obs	=	126
				LR *χ*˜^2^(10)	=	31.78
				Prob > *χ*˜^2^	=	0.0004
Log Likelihood	=	− 40.714		Pseudo *R*^2^	=	0.280
DV = SEZ	Coef	Std. Error	z	*P* > z	(95%	CI)
Urban = 1	0.06	0.46	0.12	0.90	− 0.84	0.95
Ln land area	1.28	0.98	1.31	0.19	− 0.63	3.20
Ln land area sq.	− 0.08	0.09	− 0.87	0.39	− 0.25	0.10
Ln pop. density	0.15	0.32	0.47	0.64	− 0.47	0.77
% small enterprises	0.58	0.55	1.06	0.29	− 0.50	1.66
GMS corridor = 1	0.26	0.34	0.76	0.45	− 0.41	0.93
Border = 1	1.34	0.42	3.17	0.00	0.51	2.17
North West Region	− 1.11	0.63	− 1.77	0.08	− 2.34	0.12
East & Mekong Plain Region	− 0.25	0.43	− 0.57	0.57	− 1.09	0.60
Special Region (Phnom Penh Agglom.)	1.27	0.51	2.47	0.01	0.26	2.28
Constant	− 8.03	4.15	− 1.93	0.05	− 16.16	0.11

Next, we use the generated district-level propensity scores to apply inverse probability of treatment weighting (IPTW). In brief, IPTW tries to make counterfactual inference more prominent by using the propensity scores to overweight or underweight observations that are more informative to create a quasi-experimental framework.^18^ Following best practices recommended by Austin & Stuart (2015), we apply stability-weighted IPTW in our analysis.^19^ Its calculation is depicted in Eq. 1:

1 $$w\left(x\right)=\frac{Z*P\left(Z=1\right)}{e}+\frac{\left(1-Z\right)*P\left(Z=0\right)}{\left(1-e\right)},$$

where *Z* denotes treatment, *P* (*Z* = 1) and *P* (*Z* = 0) denote the marginal probability of treatment and control observations in the overall sample, and *e* denotes a district’s propensity score. (All further uses of the abbreviation “IPTW” in this paper will refer to stability-weighted IPTW, with the exception of sensitivity tests depicted in Appendix 2, Table 14)

**Fig. 1:**
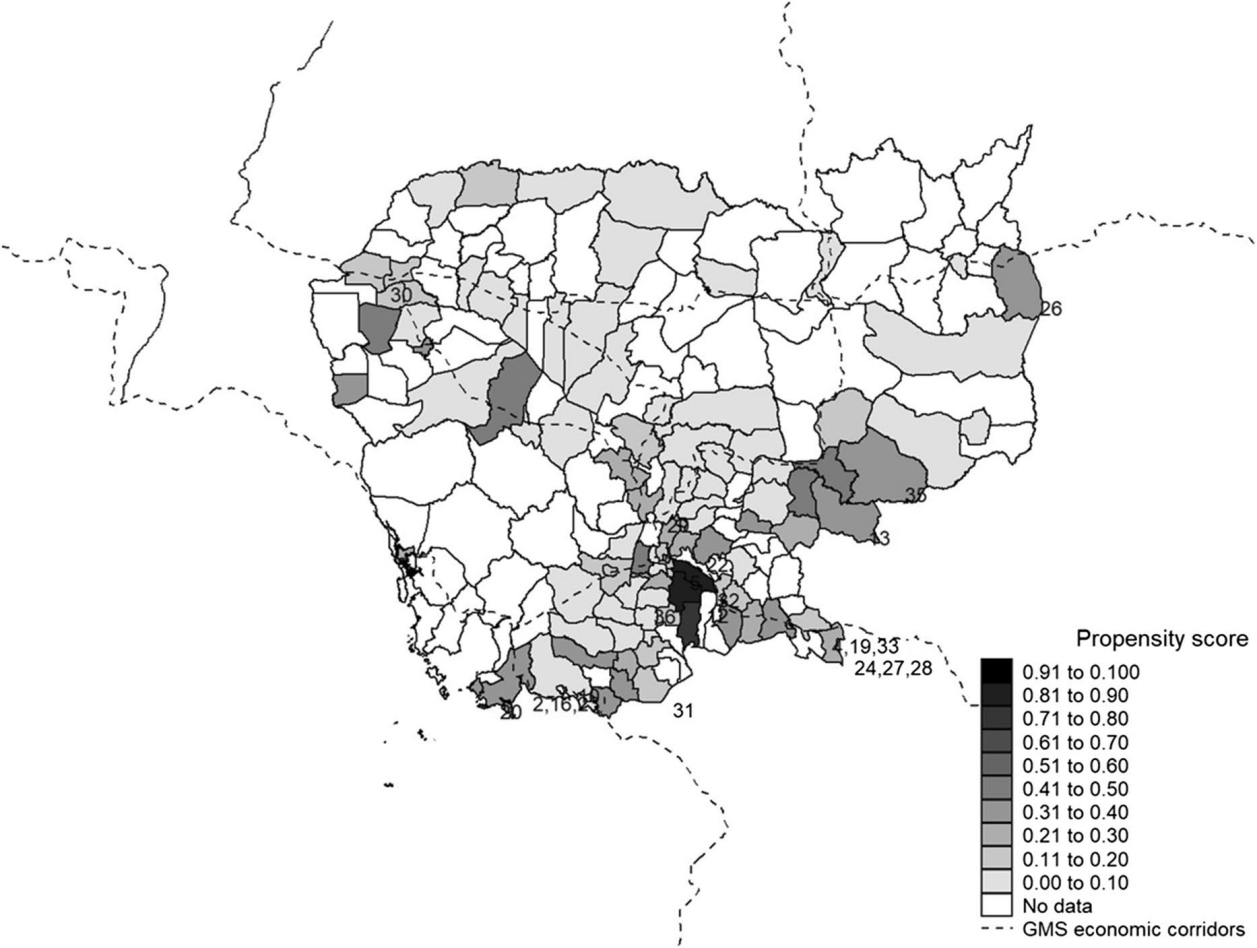
Propensities for SEZ placement across branch districts. This figure depicts propensity for SEZ placement among Cambodian districts where our research partner had branches. Treatment districts with SEZs are numbered (see Appendix A.1 for details). We run a probit regression at the district level to obtain propensity scores—estimates of the probability of SEZ presence—based on a district’s land area, population density, and broader country region, and dummy indicators for whether a district is an urban location, situated on country or ocean borders, or along the Greater Mekong Subregion economic corridor

**Fig. 2:**
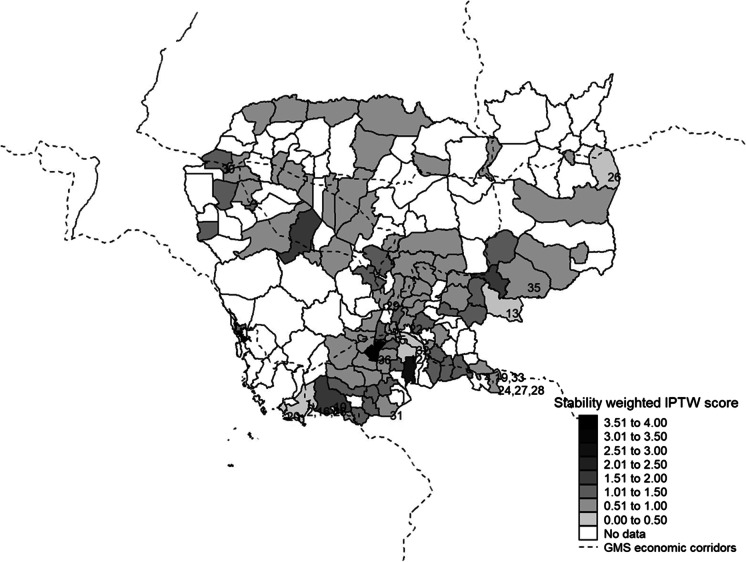
Inverse probability for treatment weights across branch districts. This figure depicts inverse probability for treatment weights (IPTW) among Cambodian districts where our research partner had branches. They are calculated using the stability weighting method detailed by Austin & Stuart (2015) and depicted in Eq. 1. Treatment districts with SEZs are numbered (see Appendix A.1 for details)

Figures 1 and 2 provide a visual depiction of the resultant propensity scores and inverse propensity for treatment scores for each district where our partner institution has branches. The districts with an SEZ that are numbered in the figures constitute our treatment group and the remaining branch districts constitute the pool for our synthetic control group. There are three takeaways. First, we can see that the SEZ districts tend to be situated around the Phnom Penh agglomeration or Northwest region, along the GMS economic corridor, or in country border areas. Second, we can observe that there are a few SEZ districts that had a relatively low propensity based on their characteristics and, conversely, several non-SEZ districts that had a high propensity for treatment. Finally, we can see that these latter treatment and control districts tend to be those that have high inverse propensity scores.

#### Assessing treatment and control group balance

We perform a series of tests in line with Caliendo & Kopeinig (2008) and Imbens & Rubin (2015) to check the appropriateness of our propensity score weighting and for meaningful differences between the established treatment and synthetic control groups. First, we conduct an omnibus test of joint orthogonality to test for balance of *district-level characteristics* after IPTW (Caliendo & Kopeinig (2008)). Specifically, after estimating propensity scores for treatment for each district where our provider had branches, we construct inverse probability treatment weights and re-estimate the same probit regression as before with the weights applied. We compare the pseudo-*R*^2^’s and *χ*^2^ statistics before and after weighting. After IPTW, there should be limited systematic differences in the distribution of covariates between each group and, therefore, the pseudo-*R*^2^ should be fairly low. Furthermore, one can also compare the likelihood ratio *χ*^2^ on the joint significance of the models’ regressors. The test should not be rejected before matching but should be rejected after matching. Comparing our post-match regression results in Table 2 with our pre-match results in Table 1, we observe that the pseudo-*R*^2^ roughly halves, and that whereas we previously rejected the null hypothesis of no joint significance in the pre-match results based on the *χ*^2^ value, we can no longer do so after the weighing.

**Table 2:** Balance test on district-level factors and SEZ implementation. This table presents coefficient estimates from a joint test of orthogonality to test for balance of district-level contextual features. We regress our district-level contextual variables on the treatment (SEZ) dummy variable and test for their joint significance. Robust standard errors

Estimation of propensity scores						
Probit Regression				# of obs	=	115
				LR *χ*˜^2^(10)	=	8.85
				Prob > *χ*˜^2^	=	0.5462
Log Likelihood	=	− 30.36		Pseudo *R*^2^	=	0.1841
DV = SEZ	Coef	Std. Error	z	*P* > z	(95%	CI)
Urban = 1	− 0.79	0.38	− 2.06	0.04	− 1.53	− 0.04
Ln land area	3.14	1.99	1.58	0.11	− 0.75	7.03
Ln land area sq.	− 0.27	0.18	− 1.49	0.14	− 0.62	0.08
Ln pop. density	0.16	0.34	0.48	0.64	− 0.51	0.84
% small enterprises	0.35	0.48	0.72	0.47	− 0.60	1.29
GMS corridor = 1	− 0.17	0.41	− 0.42	0.68	− 0.98	0.64
Border	− 0.05	0.45	− 0.10	0.92	− 0.93	0.83
North West Region	− 0.09	0.54	− 0.16	0.87	− 1.14	0.96
East & Mekong Plain Region	− 0.95	0.57	− 1.67	0.09	− 2.06	0.16
Special Region (Phnom Penh Agglom.)	− 0.59	0.60	− 0.99	0.32	− 1.76	0.58
Constant	− 10.57	5.82	− 1.82	0.07	− 21.98	0.84

Second, we test for meaningful differences in *client-*, *business-*, and *loan-level characteristics* between our treatment and our synthetic control groups. In line with Imbens & Rubin (2015), we adopt a standardized differences approach and focus on assessing the size of the differences in means rather than their statistical significance.^20^ Table 3 panels A and B summarize results from this investigation and show that the client- and loan-level characteristics generally fall within commonly suggested thresholds. There are some indications that the mean semester of loan applications was slightly later for the treatment group (the economic magnitude would equate to roughly 1.8 months), which supports controlling for time periods in later regression models. However, we interpret the overall results from our series of balance tests as supporting the suitability of the propensity score model and the IPTW method for constructing viable synthetic comparison groups for our main analysis. Furthermore, we start off our empirical analysis in Section 5 by running event study models to formally test whether patterns of business growth between treatment and control branches show signs of significant divergence in the pre-treatment period. These tests indicate that parallel trends assumption appears to hold.

**Table 3:** Summary statistics on client-, business-, and loan-level characteristics between treatment and synthetic control branch districts. This table presents summary statistics for client-, business-, and loan-level characteristics between weighted treatment and synthetic control observations. Panel A variables are run at the unique client level based on characteristics at the time of their first loan initiation in the dataset. Panel B variables are run at the loan level across all loan initiations in the dataset. The full estimation sample includes clients from 115 branches covering the period from 2011 to 2016, from which there are 9 branches in SEZ locations and 106 branches in non-SEZ locations. We estimate propensity scores for both SEZ and non-SEZ branch districts on district-level contextual factors driving “treatment”—i.e., placement of SEZs. We then apply inverse probability of treatment weighting to create synthetic counterfactual groups for the DiD estimation

Control			Treatment		Differences	
Mean Std. Dev			Mean	Std. Dev	Diff. in means	Std. diff
*Panel A. Client- and business-level variables*						
Female	0.53	0.53	0.48	1.58	− 0.05	0.040
Dependents	0.90	0.31	0.89	1.00	− 0.02	0.022
Age (under 30)	0.20	0.42	0.24	1.34	0.04	0.037
Age (31 to 40)	0.32	0.49	0.36	1.51	0.04	0.032
Age (41 to 50)	0.24	0.45	0.21	1.29	− 0.03	0.028
Age (over 50)	0.24	0.45	0.19	1.24	− 0.05	0.050
Total initial employees	1.67	2.01	1.62	6.39	− 0.06	0.012
Total initial assets (USD)	23,752	112,000	21,657	107,000	− 2095	0.019
Multiple activities	0.31	0.49	0.30	1.45	0.00	0.004
Agriculture	0.40	0.52	0.36	1.51	− 0.05	0.042
Service	0.14	0.37	0.17	1.17	0.02	0.025
Trade	0.34	0.50	0.37	1.52	0.03	0.022
Production	0.11	0.33	0.11	1.00	0.00	0.003
*Panel B. Loan-level variables*						
Total number of loan cycles	2.71	1.76	2.64	3.57	0.07	0.024
Loan amount (USD)	4980	6436	5331	19,144	351	0.025
Interest rate	24.14	7.38	24.04	23.12	− 0.10	0.006
Loan duration (years)	1.82	1.07	1.87	3.17	0.05	0.019
% loan amount collateralized	5.05	10.05	4.88	16.64	− 0.17	0.013
Semester (*Jan. 2010 = 1)	7.32	2.52	7.63	7.53	0.31	0.056
*Panel C. Employment growth outcomes*						
Absolute employment growth	− 0.03	1.77	0.02	9.18	0.05	0.008
Relative (Ln) employment growth	− 0.01	0.48	0.01	2.73	0.02	0.010
% Obs.—no change in employment	0.77	0.44	0.73	1.37	− 0.04	0.044
% Obs. —increase in employment	0.11	0.33	0.14	1.07	0.03	0.038
% Obs.—decrease in employment	0.12	0.34	0.13	1.04	0.01	0.019
*For observations with increase in employment*						
Absolute employment growth	2.03	2.31	2.24	4.92	0.21	0.054
Relative (Ln) employment growth	0.69	0.48	0.78	1.16	0.10	0.109
*For observations with decrease in employment*						
Absolute employment growth	− 2.23	2.56	− 2.29	6.89	− 0.06	0.009
Relative (Ln) employment growth	− 0.75	0.50	− 0.78	1.52	− 0.03	0.023
Observations (Panel A)	8838		757		9595	
Observations (Panels B & C)	22,666		1986		24,652	

#### Sample characteristics

Table 3 also provides an overview of sample characteristics related to borrower business growth, demographics, and loan conditions. In terms of absolute and relative employment growth, we observe that, in aggregate and at a cursory glance, mean values for either the treatment or control clients appear to be fairly minimal—between − 0.03 and 0.02 and − 0.01 and 0.01, respectively. This is consistent with stylized facts from the literature on enterprise growth, which finds that growth and growth rate distributions of enterprises among the general population tend to be heavy-tailed, particularly with respect to employment growth (Daunfeldt & Elert (2013)). In other words, the majority of enterprises in any population tend to exhibit zero or minimal employment growth, while a few will exhibit either particularly high growth or significant decline—in many settings, this results in net job creation averaging close to zero (Mead & Liedholm (1998); Davidsson et al. (2010)).

This suggests that the more interesting phenomena of enterprise growth take place in the tails of the distribution and has led the enterprise growth literature to shift attention to understanding patterns and determinants for these subsets of firms in recent years (see, for example, Goedhuys & Sleuwaegen (2010); Daunfeldt et al. (2014); Krasniqi & Desai (2016)). To explore this descriptively, we create dummy indicators for observations where client businesses experienced no change in employment, an increase in employment, or a decrease in employment between consecutive loan cycles. For each subgroup, we run separate summary statistics on the size of both employment growth and decline. We observe that a larger percentage of treatment group businesses exhibit either an increase or a decrease in employment, with the frequency of the former somewhat outweighing the latter. Furthermore, for the share of observations where businesses grew, the average size of the employment growth is larger for the treatment than the control group businesses (2.24 as opposed to 2.03 in absolute terms and 0.78 as opposed to 0.69 in relative terms). Meanwhile, for the share of observations where businesses declined, the average size of the employment decrease is also larger for the treatment than the control group businesses; however, the magnitude of the difference is noticeably smaller.

We also observe that both the weighted treatment and control groups are fairly evenly split between male and female clients and that clients are typically middle-aged and in households with other dependents. On average, businesses had between 1.62 and 1.67 employees in total (including the owner) and USD 21,700–23,800 in total assets at the time of clients’ first loan cycles. For both the treatment and the control groups, the majority of businesses were in the agriculture or trade sectors. Furthermore, the average client had close to 3 loan cycles and their typical loan size was around USD 5000–5300, with an annualized percentage rate of around 24 and loan duration of a little over one and a half years.

## Empirical analysis

Our empirical strategy is to exploit the geographic and temporal variation in Cambodia’s SEZ implementation during our study period and combine inverse probability weighting with a difference-in-differences approach. In short, the former should help mitigate selection into treatment based on observables, and the latter should further mitigate selection on unobservables that are invariant by location and time, conditional on key covariates from the extant literature.

### Event study

In Section 4.3, we provided evidence supporting the argument that our treatment and our synthetic control group are well balanced in terms of observable district- and client-level factors. However, one may be concerned that there may be unobservable factors that differ between the treatment and control districts and which may drive differences in business growth outcomes. Thus, as a first step, we investigate the key identifying assumption of difference-in-differences models—namely, that growth outcomes for clients in the treatment and the control districts would have followed parallel trends in the absence of SEZ implementation.

The validity of this identifying assumption is explored here using an event study model, which introduces lead and lagged treatment dummy indicators to test whether there are any signs of differential pre-trends. We construct the lead and lag time periods at the daily level from the specific SEZ start date and in semester blocks.^21^ For example, loan applications between 183 and 1 day prior to the SEZ start date would be considered part of the 0- to 6-month lead period (and used as the reference period for comparison) and loan applications from the SEZ start date to 183 days later would be considered part of the 0- to 6-month lag period. More specifically, for individual *i* in district *d* and at time *t*, we estimate an event study model using following form:

2 $${y}_{idt}={\theta }_{d}+{\gamma }_{t}+\sum_{j=m}^k{}{\beta }_{j}{\left(Di{D}_{idt}\times{D}_t^{j}\right)}+\chi {Controls}_{idt}+{u}_{idt}$$

where the dependent variable *y* is either (1) absolute employee growth or (2) relative (natural log) employee growth. *DiD* is an indicator variable set to 1 if district *d* had an SEZ implemented during a given study period *t*, otherwise 0. *D*^*j*^ is an indicator variable set to 1 if time period *t* is equal to *j* (where *j* can represent time periods from the minimum lead time period *m* to the maximum lagged time period of *k*), and otherwise 0. Put in other words:

$\sum_{j=m}^k{}{\beta }_{j}{\left(Di{D}_{idt}\times{D}_t^{j}\right)}$ denotes separate estimators for each time period before, during, and after SEZ implementation, whose parameters *β*_*j*_ capture whether there are any differential growth outcomes in the given time period between clients in the treatment and the control branches. Finally, *θ* and *γ* indicate branch district and time fixed effects, respectively, and *Controls* denotes a vector of client- and business-level controls, as previously described.

Under a null hypothesis of no differential pre-treatment trends, we would expect the parameter *β* on the lead treatment indicators to be zero (or statistically indistinguishable from zero) prior to the date of SEZ implementation. While not a primary focus, the coefficients on the lagged treatment indicators can also be interpreted as capturing whether the treatment effect may grow stronger or diminish over time. By design, the empirical strategy controls for factors that are time-invariant for branches across our study period (such as geographic location) and factors that affect all districts in given time periods (such as common macroeconomic shocks); that is, these factors are all purged from the estimates of *β*. As previously shown in Section 4.3, the treatment and the control groups appear relatively balanced on most client-, business-, and district-level observables. Nevertheless, to control for possible differences in individual-level characteristics between the clients in the treatment and the control branches, we introduce several relevant controls drawn from the extant literature (see discussion in Section 4.1).

**Fig. 3:**
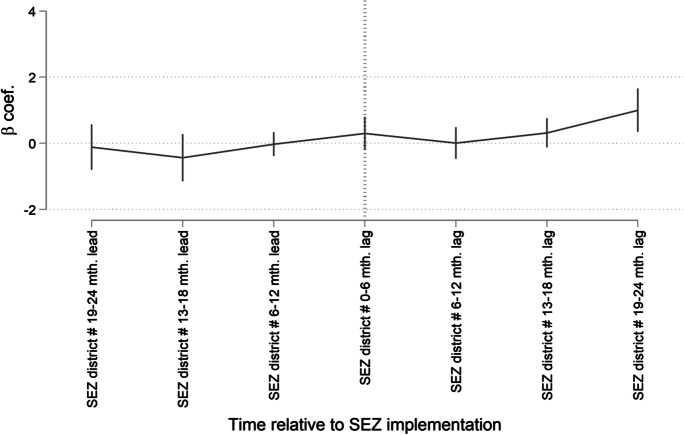
Event study model in terms of absolute employment growth. This figure depicts results from the event study model (detailed in Eq. 2), where *absolute employment growth* is the dependent variable and *βj* captures whether there are any statistically significant differences in growth outcomes, between clients in the treatment and the control branches in the periods leading up to SEZ implementation and the periods after implementation

**Fig. 4:**
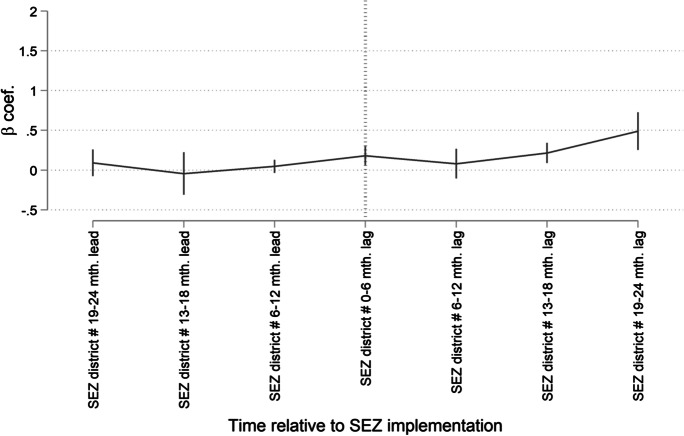
Event study model in terms of relative (Ln.) employment growth. This figure depicts results from the event study model (detailed in Eq. 2), where *relative (ln) employment growth* is the dependent variable and *βj* captures whether there are any statistically significant differences in growth outcomes, between clients in the treatment and the control branches in the periods leading up to SEZ implementation and the periods after implementation

Table 4 provides the results from the event study model for both absolute and log employment growth as main dependent variables. We observe that the differences in employment growth outcomes between the treatment and the control groups were statistically indistinguishable from one another until the period of SEZ implementation for either indicator. Thereafter, we begin to observe that employment growth for treatment group clients between consecutive loan cycles began to significantly differ in the first 0 to 6 months following implementation, with the differential growing sequentially in magnitude 13–18 months and 19–24 months later. These results are mirrored in Figs. 3 and 4, which plot the estimates of the coefficients *β*_*j*_ for the lead and the lag dummies (with 95 % confidence intervals).

**Table 4:** Event study model. This table presents coefficient estimates for an event study model measuring differences in absolute and log employment growth between SEZ (treatment) and non-SEZ (control) district clients prior to and post-SEZ implementation. The 0- to 6-month lead period serves as the comparison period (i.e., the base level). The full estimation sample includes clients from 115 branches covering the period from 2011 to 2016, of which there are nine branches in SEZ locations and 106 branches in non-SEZ locations. We estimate propensity scores for both SEZ and non-SEZ branch districts on district-level contextual factors driving “treatment”—i.e., placement of SEZs. We then apply inverse probability of treatment weighting to create synthetic counterfactual groups for the DiD estimation. Client controls include gender, household type, and age. Business controls include whether a client’s business is a micro- or small enterprise, economic sector of activity, and whether the client engages in multiple economic activities. Standard errors are clustered at the branch and semester-level and in parentheses. *** p < 0.01, ** p < 0.05, and * p < 0.1

	DV = Abs. Empl. growth	DV = Ln Empl. growth	
	(1)	(2)	
SEZ district × 19–24 mth. lead	− 0.114	0.092	
	(0.302)	(0.073)	
SEZ district × 13–18 mth. lead	− 0.436	− 0.043	
	(0.316)	(0.118)	
SEZ district × 6–12 mth. lead	− 0.026	0.048	
	(0.160)	(0.036)	
SEZ district × 0–6 mth. lag	0.303	0.180^*∗∗∗*^	
	(0.218)	(0.052)	
SEZ district × 6–12 mth. lag	0.009	0.081	
	(0.212)	(0.083)	
SEZ district × 13–18 mth. lag	0.316	0.216^*∗∗∗*^	
	(0.195)	(0.056)	
SEZ district × 19–24 mth. lag	1.002^*∗∗∗*^	0.489^*∗∗∗*^	
	(0.290)	(0.104)	
*Additional controls:*			
Branch fixed effects	Yes	Yes	
Semester fixed effects	Yes	Yes	
Client controls	Yes	Yes	
Business controls	Yes	Yes	
Loan controls	No	No	
Observations	6901	6901	
*R*^2^	0.073	0.074

### Generalized model

Results from the event study model are consistent with our first hypothesis, namely that the SEZ implementation has a positive effect on employment growth for our research partner’s entrepreneurial clients. To estimate the effect magnitude during our study period and to run sensitivity tests, we next use a generalized difference-in-differences model of the following form:

3 $${y}_{idt}={\theta }_{d}+{\gamma }_{t}+\beta Di{D}_{idt}+\chi {Controls}_{idt}+{u}_{idt}$$

where, rather than using separate interactions for each period, the model has a single estimator, *DiD*, which denotes whether a district *d* has had SEZ implementation in time *t* (that is, an interaction on treatment and the post-treatment period). The parameter *β* is our main coefficient of interest in this simple specification. All other variables are as previously described.

In Table 5, we summarize the results of different model specifications, which include (a) a baseline model with district and semester fixed effects and our main difference-in-differences estimator, (b) adding client-level controls, (c) adding business-level controls, and (d) further controlling for loan-level characteristics. The third specification (c) is our preferred specification and parallels the prior event study models. The inclusion of loan-level characteristics in the specification (d) may understandably be an example of “bad controls” as they are more likely to be endogenous; their inclusion is more to demonstrate the general robustness of results.

**Table 5:** Generalized model—the impact of SEZ implementation on employment growth in clients’ businesses. This table presents coefficient estimates for a generalized difference-in-difference model measuring the effect of SEZ implementation on absolute and relative (log) annualized employment growth. *DiD estimator* denotes whether a district *d* had SEZ implementation in time *t* (i.e., an interaction on being in the treatment group and in the post period). The estimation sample includes clients from 115 branches covering the period from 2011 to 2016, from which there are 9 branches falling in the treatment group and 106 falling in the control. We estimate propensity scores for both treatment and control branch districts on district-level factors driving placement of SEZs. We then apply inverse probability of treatment weighting to create synthetic counterfactual groups for the DiD estimation. We estimate different model specifications which include a (a) baseline model with just district and time (semester) fixed effects and our main difference-in-differences estimator, (b) including borrower-level controls, (c) further for business-level characteristics, and (d) also adding in loan characteristics. Client controls include gender, household type, and age. Business controls include whether a client’s business is a micro- or small enterprise, economic sector of activity, and whether the client engages in multiple economic activities. Loan characteristics include whether it is a first loan cycle or not, ln. loan amount (USD), interest rate, loan duration, and percent of loan amount collateralized. Standard errors are clustered at the branch and semester-level and in parentheses. *** p < 0.01, ** p < 0.05, and * p < 0.1

	DV = Abs. employment growth				DV = Rel. (Ln) employment growth			
	(1)	(2)	(3)	(4)	(5)	(6)	(7)	(8)
*DiD* estimator	0.393 ∗ ∗ ∗	0.400 ∗ ∗ ∗	0.353 ∗ ∗ ∗	0.359 ∗ ∗ ∗	0.144 ∗ ∗ ∗	0.147 ∗ ∗ ∗	0.137 ∗ ∗ ∗	0.140 ∗ ∗ ∗
	(0.000)	(0.002)	(0.010)	(0.005)	(0.000)	(0.000)	(0.001)	(0.001)
*Additional controls:*								
Branch fixed effects	Yes	Yes	Yes	Yes	Yes	Yes	Yes	Yes
Semester fixed effects	Yes	Yes	Yes	Yes	Yes	Yes	Yes	Yes
Borrower controls	No	Yes	Yes	Yes	No	Yes	Yes	Yes
Business controls	No	No	Yes	Yes	No	No	Yes	Yes
Loan characteristics	No	No	No	Yes	No	No	No	Yes
Observations	6901	6901	6901	6901	6901	6901	6901	6901
*R*^2^	0.034	0.035	0.062	0.064	0.054	0.054	0.064	0.066

In columns 5 through 8 of Table 5, we observe that the implementation of SEZs increased employment growth in relative terms by 13.7 to 14.7 % between loan cycles for clients in SEZ districts with respect to clients in control districts. In absolute terms, the *DiD* estimators in columns 1 through 4 indicate between a 0.35 and a 0.40 absolute increase in the number of employees between clients in the treatment and the control districts before and after the SEZ implementation. Taking into consideration the mean initial business size for clients from our sample of roughly 1.7 total employees (see Table 3), an alternative way of interpreting the absolute economic magnitude of the effects on clients is that every 10 client businesses in the SEZ districts started out with approximately 17 employees *combined* and ended up with roughly 3 to 5 more additional employees *combined* compared to similar client businesses in the control districts, following SEZ implementation. It is worth recalling that this represents the average difference in employment growth between treatment and control clients typically around one year from a given loan cycle. We can further observe that the additional array of controls improves the predictive power of our models but that our main results are generally quite robust to their inclusion. For example, our *DiD* estimator remains significant at the 5 % level in all specifications. The coefficient marginally changes with the inclusion of client controls and business-level controls (columns 2, 3, 6, and 7), and loan characteristics (columns 4 and 8), but remains in a similar range.^22^

In Appendix 3, we also present a number of sensitivity tests to demonstrate that the main results are fairly insensitive to changes in various aspects of the construction of the analysis, including using alternative methods of propensity score construction to determine the synthetic control group and changing the time-unit level of analysis—for example, from semester to annual. Furthermore, we present suggestive evidence that the main result is unlikely to be driven by differences in client attrition rates between treatment and control districts.

One factor that does appear to have an important influence on results—both in this study and in empirical enterprise growth studies more generally—is the inclusion or exclusion of outliers. We observe, for example, that the economic and statistical significance of the main result does diminish as an increasing amount of the tails are removed from the sample, roughly halving after the top and bottom five percentiles have been winsorized. While the overall results still stand, the results are in line with the literature in highlighting that the majority of business expansion (and contraction) occurs at the tails of the distribution. That is, a smaller but important share of entrepreneurial clients has a large influence on results.

### Mechanisms and differential enterprise growth outcomes

To identify the key mechanisms through which SEZs helped improve business environments, we set up additional empirical specifications to test which types of borrowers saw the greatest benefits from SEZ implementation and under which contextual settings. We start by investigating those client- and district-level factors that are linked to differential effects on MSEs’ business growth when their access to finance was combined with being located in an SEZ. Subsequently, we investigate the credit provision channel by testing how SEZ implementation affected credit demand and supply, and by linking the observed changes to different enterprise growth outcomes.

#### Enterprise-level factors driving differential enterprise growth outcomes

We begin by exploring the paper’s Hypotheses 2a to 2c, which focus on client- and business-level factors that are linked to differential growth outcomes. To do so within the framework of our previous analysis, we introduce these factors as interaction terms with our main difference-in-differences estimator and rerun our generalized model (Eq. 3) across our full country sample. The coefficients of interest for these models are on the additional interaction term, where a significant coefficient indicates that the factor moderates the intensity of the treatment effect. Table 6 reports both main SEZ effects and the differential effects when interacted with our client-level factors of interest.

**Table 6:** Client-level factors affecting intensity of treatment effects. The table presents coefficient estimates for a generalized difference-in-differences model measuring the effect of SEZ implementation on client business growth. *DiD estimator* denotes whether a district *d* had SEZ implementation in time *t* (i.e., an interaction on being in the “treatment” group and in the post- “treatment” period). We explore differential treatment effects driven by client-level factors through the inclusion of an additional interaction term. All models also include the previously established controls, including other client demographics (age, dependents), business characteristics (multiple business activities), branch fixed effects, and time fixed effects. Standard errors are clustered at the branch and semester-level and in parentheses. *** p < 0.01, ** p < 0.05, and * p < 0.1

	DV = Abs. employment growth						DV = Rel. (Ln) employment growth					
	(1)	(2)	(3)	(4)	(5)	(6)	(7)	(8)	(9)	(10)	(11)	(12)
*Main effects*												
*DiD* estimator	0.352^∗∗∗^	0.349^∗∗∗^	0.337^∗∗∗^	0.337^∗∗∗^	0.387^∗∗∗^	0.429^∗∗∗^	0.129^∗∗∗^	0.125^∗∗∗^	0.134^∗∗∗^	0.122^∗∗∗^	0.147^∗∗∗^	0.138^∗∗∗^
	(0.068)	(0.072)	(0.061)	(0.077)	(0.080)	(0.101)	(0.027)	(0.030)	(0.032)	(0.024)	(0.027)	(0.043)
Small business	− 1.428^∗∗∗^	− 1.399^∗∗∗^	− 1.398^∗∗∗^	− 1.399^∗∗∗^	− 1.400^∗∗∗^	− 1.398^∗∗∗^	− 0.213^∗∗∗^	− 0.213^∗∗∗^	− 0.213^∗∗∗^	− 0.213^∗∗∗^	− 0.215^∗∗∗^	− 0.212^∗∗∗^
	(0.253)	(0.250)	(0.249)	(0.250)	(0.249)	(0.250)	(0.039)	(0.038)	(0.038)	(0.038)	(0.041)	(0.038)
Agriculture	0.066	0.059	0.060	0.062	0.058	0.058	− 0.004	− 0.005	− 0.004	− 0.003	− 0.007	− 0.004
	(0.333)	(0.333)	(0.333)	(0.333)	(0.333)	(0.332)	(0.120)	(0.120)	(0.120)	(0.120)	(0.119)	(0.120)
Service	0.180	0.176	0.171	0.178	0.174	0.174	− 0.003	− 0.003	− 0.002	− 0.003	− 0.008	− 0.003
	(0.332)	(0.332)	(0.332)	(0.332)	(0.332)	(0.331)	(0.119)	(0.119)	(0.119)	(0.119)	(0.118)	(0.119)
Trade	0.156	0.154	0.154	0.154	0.152	0.152	0.008	0.008	0.008	0.007	0.009	0.007
	(0.327)	(0.327)	(0.328)	(0.327)	(0.327)	(0.326)	(0.118)	(0.118)	(0.118)	(0.118)	(0.117)	(0.118)
Production	0.087	0.082	0.083	0.084	0.093	0.080	− 0.017	− 0.017	− 0.017	− 0.016	− 0.013	− 0.017
	(0.345)	(0.346)	(0.346)	(0.346)	(0.346)	(0.345)	(0.124)	(0.124)	(0.124)	(0.123)	(0.122)	(0.124)
Female	− 0.010	− 0.009	− 0.008	− 0.009	− 0.009	0.000	− 0.007	− 0.007	− 0.007	− 0.007	− 0.012	− 0.005
	(0.038)	(0.038)	(0.037)	(0.038)	(0.038)	(0.042)	(0.011)	(0.011)	(0.011)	(0.011)	(0.010)	(0.012)
*Client-level interaction effects*												
Small business = 1 X *DiD* estimator = 1	1.415^*∗∗∗*^ (0.244)						0.031 (0.039)					
Agriculture = 1 × *DiD* estimator = 1		0.016 (0.069)						0.013 (0.026)				
Service = 1 × *DiD* estimator = 1			0.130 (0.132)						− 0.038 (0.039)			
Trade = 1 × *DiD* estimator = 1				0.039 (0.064)						0.016 (0.028)		
Production = 1 × *DiD* estimator = 1					− 0.259 (0.197)						− 0.027 (0.035)	
Female × *DiD* estimator = 1						− 0.160 (0.128)						− 0.019 (0.051)
Observations	6901	6901	6901	6901	6901	6901	6901	6901	6901	6901	6901	6901
*R*^2^	0.064	0.063	0.063	0.063	0.064	0.064	0.063	0.063	0.063	0.063	0.066	0.063

First, we test whether there were signs of differential effects observed between small- and microenterprise clients due to SEZ implementation (Hypothesis 2a). This is in line with the suggestion of Beck (2015) that moving up the firm ladder to focus on small rather than micro enterprises might have more potential to be transformative and create jobs. In columns 1 and 7, we observe positive coefficients for the interaction between small businesses and the *DiD* estimator of large economic sizes, albeit statistically significant in absolute terms only. Nevertheless, this finding is more striking when noting that we do not observe a significant relationship with employment growth for small enterprise clients outside of SEZ areas but rather generally negative relationships. It is important to note that coefficients for the *DiD* main effects in columns 1 and 7 remain both statistically and economically significant. This generally implies that the increased business growth due to SEZ implementation generally benefited micro enterprise clients as well.

Next, we explore whether there were differential effects from SEZ implementation for clients whose businesses were in different sectors of economic activity (Hypothesis 2b). In the Cambodian case, the SEZs have been heavily oriented around developing regional linkages for agricultural and agriculture processing firms and global linkages for manufacturing (particularly garments) firms. Differentiating our analysis by sectors of activity and taking the results depicted in Table 6 columns 2–5 and 8–11 as a whole, we do not find strong evidence that higher growth was heavily concentrated within particular sector(s) of activity, across the full country sample.

Finally, we also explore differential effects for female versus male clients (Hypothesis 2c). As noted previously, women in Cambodia have historically faced barriers to equal economic, social, and political participation. This is driven in part by long-standing behavior norms for women that have traditionally created gender inequality in education and occupational choice (UNIFEM (2004); Ven & Pham (2022)). As Cambodia’s SEZ policies do not appear to target any of the underlying structural reasons hindering women’s entrepreneurial opportunities, our *a priori* expectation is that male-owned enterprise borrowers will continue to be relatively better positioned to benefit from SEZ implementation in terms of growth opportunities. In Table 6, columns 6 and 10, we see some signs of negative interactions between the *DiD* estimator and the dummy variable for female clients, albeit the effect is not found to be significant. In other words, across our full sample, employment growth driven by SEZ implementation was not notably more pronounced for male rather than female clients, albeit there are some suggestive signs of a negative differential. It is worth noting that the main effect size from the SEZ implementation outweighs the (suggestive) negative differential for women and would still lead to a net positive impact. It thus appears that clients of both genders were able to significantly improve their businesses’ growth due to the SEZ implementation.

#### Cross-sectional heterogeneity: country border vs. interior locations

Our analysis so far aggregates MSEs across a variety of Cambodian SEZs and clients in different contexts, which may lead to conflicting and thus insignificant interactions effects. In practice, the Cambodian SEZs as described in Section 3 can be distinguished into two groups. The first group includes SEZs situated near the country borders with Thailand and Vietnam, which are posited to have benefited from the improved infrastructure (the GMS corridor) and expedited cross-border customs and bureaucracy, giving them improved access to the markets in their more developed neighbors. Given the cheaper land and labor on the Cambodian side, this is expected to have created opportunities to supply local regional markets in Southeast Asia with lower value-added goods and services—e.g., agricultural goods, light manufacturing, and tourism. The second group includes SEZs in the interior districts (many clustered in the outskirts around Phnom Penh) and along ocean borders, which have traditionally been orientated toward more global export markets. In practice, the activities in these SEZs are heavily concentrated in the garment sector. We posit that these different SEZ types may provide growth opportunities for different types of MSEs. To test this empirically, we thus split the sample—i.e., both treatment and control districts—between country border districts versus interior and ocean border districts and rerun the analysis using the same interaction specifications as before.

In Table 7, we restrict our sample to just country border districts and find greater evidence that the benefits of SEZ implementation in these settings was concentrated among certain borrower types. In particular, we see strong indication that borrowers in agricultural activities saw considerably increased employment growth in both absolute and relative terms, due to the onset of the SEZs. As noted in Section 2, this is consistent with anecdotal reports about improved agricultural trade driven by the GMS corridors. We also see signs that both small business enterprises and those in the services sector saw significantly increased growth in absolute terms. There is also a stronger gender differential revealed where men-owned businesses see much higher growth (in both statistical and economic terms) relative to women-owned businesses following SEZ implementation. This is consistent with prior evidence on constraints in Cambodia for women entrepreneurs and with general findings from the literature on access to finance (McKenzie et al. (2008); Banerjee et al. (2015a); Beck (2015)), which have documented that many interventions are less successful for women than for men, given intrahousehold and other constraints holding back women. This implies that such interventions have to further take into account these specific constraints faced by women to be successful at promoting inclusive outcomes. These combined results indicate that there are differential patterns at border districts and suggest that the SEZ implementation there tended to favor certain types of businesses.

**Table 7:** Client-level factors affecting intensity of treatment effects, sample restricted to country border districts. The table presents coefficient estimates for a generalized difference-in-differences model measuring the effect of SEZ implementation on client business growth. *DiD estimator* denotes whether a district *d* had SEZ implementation in time *t* (i.e., an interaction on being in the “treatment” group and in the post- “treatment” period). We restrict the sample to districts on the country land borders (with Thailand and Vietnam). We explore differential treatment effects driven by client-level factors through the inclusion of an additional interaction term. All models also include the previously established controls, including other client demographics (age, dependents), business characteristics (multiple business activities), branch fixed effects, and time fixed effects. Standard errors are clustered at the branch and semester-level and in parentheses. *** p < 0.01, ** p < 0.05, and * p < 0.1

	DV = Abs. employment growth						DV = Rel. (Ln) employment growth					
	(1)	(2)	(3)	(4)	(5)	(6)	(7)	(8)	(9)	(10)	(11)	(12)
*Main effects*												
*DiD* estimator	0.323^∗∗^	0.326^∗∗^	0.264^∗∗^	0.323^∗∗^	0.423^∗∗^	0.506^∗∗∗^	0.137^∗∗∗^	0.136^∗∗∗^	0.142^∗∗∗^	0.140^∗∗∗^	0.145^∗∗∗^	0.179^∗∗∗^
	(0.123)	(0.123)	(0.109)	(0.128)	(0.166)	(0.160)	(0.045)	(0.045)	(0.049)	(0.044)	(0.051)	(0.042)
Small business	− 1.157^∗∗^	− 1.091^∗∗^	− 1.098^∗∗^	− 1.101^∗∗^	− 1.102^∗∗^	− 1.097^∗∗^	− 0.140^∗∗^	− 0.138^∗∗^	− 0.142^∗∗^	− 0.142^∗∗^	− 0.142^∗∗^	− 0.141^∗∗^
	(0.506)	(0.495)	(0.489)	(0.491)	(0.488)	(0.492)	(0.061)	(0.058)	(0.058)	(0.058)	(0.058)	(0.058)
Agriculture	0.040	0.019	0.016	0.027	0.024	0.032	− 0.017	− 0.019	− 0.015	− 0.016	− 0.016	− 0.015
	(0.258)	(0.258)	(0.262)	(0.255)	(0.253)	(0.249)	(0.101)	(0.102)	(0.100)	(0.101)	(0.101)	(0.099)
Service	0.187	0.174	0.148	0.175	0.164	0.185	− 0.018	− 0.018	− 0.015	− 0.018	− 0.018	− 0.015
	(0.266)	(0.264)	(0.270)	(0.265)	(0.261)	(0.257)	(0.106)	(0.107)	(0.108)	(0.108)	(0.106)	(0.105)
Trade	0.126	0.122	0.114	0.119	0.108	0.126	− 0.002	− 0.001	− 0.002	− 0.002	− 0.003	− 0.001
	(0.260)	(0.258)	(0.264)	(0.257)	(0.255)	(0.252)	(0.098)	(0.099)	(0.098)	(0.098)	(0.098)	(0.097)
Production	0.074	0.061	0.057	0.062	0.090	0.072	− 0.048	− 0.048	− 0.047	− 0.048	− 0.045	− 0.045
	(0.294)	(0.293)	(0.299)	(0.295)	(0.294)	(0.287)	(0.115)	(0.115)	(0.114)	(0.116)	(0.117)	(0.113)
Female	− 0.027	− 0.023	− 0.024	− 0.024	− 0.024	− 0.000	− 0.014	− 0.014	− 0.014	− 0.014	− 0.014	− 0.009
	(0.073)	(0.073)	(0.073)	(0.073)	(0.073)	(0.083)	(0.023)	(0.023)	(0.023)	(0.023)	(0.023)	(0.026)
*Client-level interaction effects*												
Small business = 1 × *DiD* estimator = 1	1.138^*∗∗*^ (0.489)						− 0.043 (0.061)					
Agriculture = 1 × *DiD* estimator = 1		0.433^*∗∗∗*^ (0.142)						0.211^*∗∗∗*^ (0.031)				
Service = 1 × *DiD* estimator = 1			0.276^*∗*^ (0.162)						− 0.023 (0.052)			
Trade = 1 × *DiD* estimator = 1				0.010 (0.104)						− 0.005 (0.042)		
Production = 1 × *DiD* estimator = 1					− 0.383 (0.326)						− 0.032 (0.083)	
Female × *DiD* estimator = 1						− 0.401^*∗∗*^ (0.165)						− 0.095^*∗∗*^ (0.038)
Observations	2185	2185	2185	2185	2185	2185	2185	2185	2185	2185	2185	2185
*R*^2^	0.054	0.053	0.053	0.053	0.053	0.054	0.056	0.057	0.056	0.056	0.056	0.057

Meanwhile, it is worth noting that we generally see less evidence of differential growth patterns in the interior and ocean border districts, as depicted in Appendix Table 21. A possible explanation for this may have to do with the fact that economic activity in those settings is heavily orientated toward the garment industry, which generally entails larger and more capitalized firms. As our dataset only contains MSEs (with up to 50 employees), we may be missing many of the firms that saw greatest change in growth in those settings following SEZ creation.

Overall, our findings indicate that at an aggregate level, there were smaller signs of differential effects of SEZ implementation on MSE employment growth. However, in country border districts, there was a particular segment of MSE borrowers that was able to disproportionately benefit from access to microcredit, in terms of expanding their enterprises.

#### District- and SEZ-level contextual factors driving differential enterprise growth outcomes

We similarly explore the paper’s Hypotheses 3a to 3d on territorial-level factors that are linked to differential effects from SEZs. In line with Frick et al. (2018) and based on availability of relevant data, we introduce a number of district- and SEZ-level interaction terms with the *DiD* estimator to test if and to what extent the factors are shown to significantly moderate the intensity of SEZ-induced employment growth in the context of Cambodian microcredit clientele.

First, we test the stylized fact that higher-growth firms are disproportionately concentrated in areas with higher population density and growth (Hypothesis 3a), which has been found in a large body of studies—e.g., Liedholm (2002); Acs & Mueller (2008); and Coad (2009). As highlighted by Stam (2010), the mechanisms explaining this could include improved access to markets and customers as well as to the inputs required (for example, capital, labor/employees, suppliers) to produce goods or services. To do so, we construct dummy indicators for clients that are above (versus below) the median in terms of population density or annualized population growth. We find, as shown in Table 8 columns 1 and 6, a significant positive interaction between SEZ implementation and areas with higher population density on clients’ employment growth. This difference is economically quite large, translating into a greater increase of 2.1 employees in absolute terms and 44.5 % higher growth in relative terms. We find similar signs of a significant positive interaction between SEZ implementation and areas with higher population growth on clients’ employment growth, albeit only significant in relative terms (in column 7).

**Table 8:** District- and SEZ-level factors affecting intensity of treatment effects. The table presents coefficient estimates for a generalized difference-in-differences model measuring the effect of SEZ implementation on client business growth. *DiD estimator* denotes whether a district *d* had SEZ implementation in time *t* (i.e., an interaction on being in the “treatment” group and in the post- “treatment” period). We explore differential treatment effects driven by district- and SEZ-level factors through the inclusion of an additional interaction term. The estimation sample includes clients from 115 branches covering the period from 2011 to 2016, of which there are nine branches in the treatment group and 106 in the control. We estimate propensity scores for both treatment and control branch districts on district-level contextual factors driving placement of SEZs. We then apply inverse probability of treatment weighting to create synthetic counterfactual groups for the DiD estimation. All models also include the previously established controls, including client demographics, business characteristics, branch fixed effects, and time fixed effects. Standard errors are clustered at the branch and semester-level and in parentheses. *** p < 0.01, ** p < 0.05, and * p < 0.1

	DV = Abs. employment growth					DV = Rel. (Ln) employment growth				
	(1)	(2)	(3)	(4)	(5)	(6)	(7)	(8)	(9)	(10)
*DiD* estimator	0.324^∗∗∗^	0.311^∗∗∗^	0.316^∗∗∗^	0.349^∗∗∗^	0.647	0.132^∗∗∗^	0.109^∗∗∗^	0.111^∗∗∗^	0.110^∗∗∗^	0.102
	(0.058)	(0.064)	(0.062)	(0.094)	(0.707)	(0.024)	(0.021)	(0.021)	(0.028)	(0.188)
High pop. density district × *DiD* estimator = 1	2.117^∗∗∗^					0.445^∗∗∗^				
	(0.082)					(0.033)				
High pop. growth district × *DiD* estimator = 1		0.093					0.049^∗∗^			
		(0.100)					(0.021)			
Country border × *DiD* estimator = 1			0.044					0.037^∗∗^		
			(0.053)					(0.015)		
SEZ start—1st half study period × *DiD* estimator = 1				-0.005					0.041^∗^	
				(0.080)					(0.021)	
Close distance btw. SEZ and branch × *DiD* estimator = 1					-0.313					0.036
					(0.706)					(0.188)
Observations	6901	6901	6901	6901	6901	6901	6901	6901	6901	6901
*R*^2^	0.063	0.064	0.062	0.062	0.062	0.064	0.066	0.064	0.064	0.064

Next, in line with our analysis in Section 5.3.2, we formally test whether there are differential impacts from SEZs for clients located in country border versus interior/ocean districts (Hypothesis 3b). This is motivated by the dichotomy in Cambodia’s SEZs described above. In column 8, we similarly find general support that the effect on relative employment growth of SEZ implementation was also significantly higher in country border districts relative to interior/ocean districts.

Second, in relation to SEZ characteristics, we test for interaction effects for SEZs that had earlier versus later start dates in our study period (as a proxy for zone maturity) and for those in closer physical proximity to the service provider branch. For simplicity, these factors are captured as two dummy variables that are set to 1, respectively, for locations whose SEZ started in the first half of the study period and for locations where the distance between SEZ and branch locations are in the bottom 50th percentile. The first of these hypotheses (Hypothesis 3c) follows the findings of Farole (2011), who does a cross-country and long-term comparison of SEZs, and finds that many successful SEZs have required an incubation period, with slower growth in the initial years that then grows exponentially once significant time passes—i.e., as SEZs become increasingly “mature.” In column 9 of Table 8, we find some supporting evidence in favor of this hypothesis, as client businesses in the older SEZs are found to exhibit significantly higher average relative employment growth. The latter hypothesis (Hypothesis 3d) tests our general intuition that the benefits to the local economy from the SEZ, if any, will be more likely to occur in closer proximity to the zone itself. In practice, we observe mixed and largely insignificant results for the interaction between close proximity branches and SEZs (in columns 5 and 10). However, it is worth clarifying that the constructed variable only captures the distance between the branch and SEZ locations rather than between a client’s actual business location and SEZs specifically. As such, it may be a somewhat less precise measure, compared to our variable capturing the maturity of SEZs.

Taken together, our findings highlight the importance of broader territorial conditions that may be conducive to business growth. In our study setting and given the context of Cambodia’s SEZs, we observe signs that business growth was most heavily concentrated in areas where clients had access to dynamic labor markets and where the SEZ onset was most likely to facilitate access to new regional markets.

#### SEZ implementation and credit provision

Finally, we study how SEZ implementation impacted both credit demand and supply to understand whether they may provide additional channels for explaining the improved business outcomes for the lender’s borrowers. On the one hand, the onset of SEZs may have led to systematic differences in the amount of demand and the composition of incoming borrowers (presumably in ways that favor higher-growth enterprises). On the other hand, the SEZs may have also led affected branches to adjust their loan terms and conditions in ways that may have been more favorable to their clients’ enterprise growth. Each scenario alone or their combination may also help provide plausible explanations for the observed improvement in client outcomes.

To examine this, we set up an alternative difference-in-difference specification at the branch level, as depicted in Eq. 4 below:

4 $${y}_{idt}={\theta }_{d}+{\gamma }_{t}+\beta Di{D}_{idt}+{u}_{idt}$$

where the dependent variable *y* denotes several demand and supply-side indicators, which are now calculated at the branch-level for branch *i* during period *t*. Specifically, the demand-side indicators include the log change in the total number of loans requested, and the share of borrowers who are first time borrowers, small enterprises, agricultural sector, service sector, trade sector, or manufacturing sector, respectively. Meanwhile, the supply-side indicators include the log change in total loan volume disbursed and log change in the average loan amount, interest rate, maturity, and percent of loan amount collateralized. As before, *DiD* is an indicator variable set to 1 if district *d* had an SEZ implemented during a given period *t*, otherwise 0 and where *θ* and *γ* indicate branch district and time fixed effects, respectively. The DiD coefficient, *β*, measures how borrower demand and composition and the average terms on new loans from branches dynamically changed when SEZs were implemented.

The results from the demand-side analysis are provided in Table 9 and generally do not suggest that there were notable changes in the demand for credit due to SEZ implementation. That is, we do not observe any statistically or economically significant changes in the number of loans being demanded or the types of incoming borrowers following SEZ implementation, such as the share of borrowers that are first-time clients, small enterprise owners, or whose economic activity are in the agricultural, service, trade, or manufacturing sectors, respectively. By contrast, the results from the supply-side analysis in Table 10 provide indication that there were improvements in the loan terms and conditions offered to borrowers in areas where SEZs were implemented. In particular, we find that overall loan volume in the affected branches increases significantly in absolute terms—by around 40,000 USD per SEZ-based branch—and that average loan amounts are 18.9 % higher. (These seem linked since we do not find significant increases in the total number of loans being disbursed in Table 9.) Moreover, we observe that collateral requirements decrease by around 16.6 %.

**Table 9:** SEZ implementation and credit demand. This table presents coefficient estimates for a generalized difference-in-difference model measuring the effect of SEZ implementation on credit demand indicators. The heading of each column indicates the dependent variable in each specification. These include the following: (1) log change in the total number of loans requested and the percentage of incoming borrowers who are (2) first time borrowers, (3) small enterprises, (4) agricultural sector, (5) service sector, (6) trade sector, and (7) manufacturing sector, for a given branch and time period. As before, *DiD* is an indicator variable set to 1 if district *d* had an SEZ implemented during a given period *t*, otherwise 0. All models include branch district and time fixed effects. "Standard errors are clustered at the branch and semester-level and in parentheses. *** p < 0.01, ** p < 0.05, and * p < 0.1

	Total number of loans (Ln)		% Extensive margin	% Small enterprises	% Agriculture	% Service	% Trade	% Manufacturing
	(1)		(2)	(3)	(4)	(5)	(6)	(7)
*DiD* estimator	0.046		0.015	-0.028	0.007	0.020	-0.039	0.016
	(0. 178)		(0.023)	(0.021)	(0.097)	(0.064)	(0.087)	(0.081)
*Additional controls:*								
Branch fixed effects		Yes	Yes	Yes	Yes	Yes	Yes	Yes
Semester fixed effects		Yes	Yes	Yes	Yes	Yes	Yes	Yes
Observations	992		992	992	992	992	992	992
*R*^2^	0.887		0.899	0.823	0.759	0.557	0.606	0.430

**Table 10:** SEZ implementation and credit supply. This table presents coefficient estimates for a generalized difference-in-difference model measuring the effect of SEZ implementation on credit supply indicators. The heading of each column indicates the dependent variable in each specification. These include the following: (1) total loan volume disbursed and logarithmic change in average (2) loan amount, (3) interest rate, (4) maturity, and (5) percent of loan amount collateralized. The model is aggregated and estimated at the branch-time period level. As before, *DiD* is an indicator variable set to 1 if district *d* had an SEZ implemented during a given period *t*, otherwise 0. All models include branch district and time fixed effects. Standard errors are clustered at the branch and semester-level and in parentheses. *** p < 0.01, ** p < 0.05, and * p < 0.1

	Absolute loan volume (USD)	Ln avg. loan amount	Ln avg. interest rate	Ln avg. maturity	Ln avg. % collateralized
	(1)	(2)	(3)	(4)	(5)
*DiD* estimator	39,128^*∗∗∗*^	0.189^*∗*^	-0.019	-0.118	-0.166^*∗∗*^
	(8793)	(0.088)	(0.019)	(0.102)	(0.074)
*Additional controls:*					
Branch fixed effects	Yes	Yes	Yes	Yes	Yes
Semester fixed effects	Yes	Yes	Yes	Yes	Yes
Observations	992	992	992	992	992
*R*^2^	0.740	0.721	0.835	0.768	0.612

Given our data, we are not able to readily disentangle whether the increased loan amounts and reduced collateral requirements are because the incoming borrowers are deemed more creditworthy (or less ex ante risky) or because the affected branches of the lender react positively to SEZ implementation as likely to improve prospects for their borrowers. However, the lack of any observable shifts in borrower composition from our demand-side analyses in Table 9 provides greater support to the latter hypothesis. That is, we interpret the combined results as a supply-side shock from the lender where affected branches reacted to the improving business opportunities by providing better loan terms to their borrowers.

#### Credit provision and enterprise growth outcomes

Finally, we test whether the combination of better quality of financial access and SEZ implementation help predict differential growth patterns for entrepreneurial borrowers (Hypotheses 4a and 4b). To do so, we introduce loan terms and length of borrower-lender relationships as interaction terms with our main difference-in-differences estimator and rerun our generalized model (Section 5.2, Eq. 3). This mirrors the borrower-loan level model specifications used in our analyses for Table 6 to Table 8. The coefficients of interest for these models are on both the main effects of the financial access quality indicators and the interaction term between these indicators and SEZ implementation, where a significant coefficient indicates it moderates the intensity of the treatment effect.

The results are reported in Table 11 and provide strong support to the hypothesis that the combination of SEZ implementation with improved loan terms for borrowers led to particularly higher employment growth. We find that in districts where SEZs were implemented, a 10% increase in loan amount for borrowers roughly translates to an 11% greater relative increase in employment and a 10% decrease in the loan amount collateralized would translate into a 7% relative increase in employment. We also see similar positive relationships between decreased interest rates and longer loan duration in predicting employment growth, albeit give them less focus since we did not observe as strong signs in Table 10 that they changed on average at the branch level due to SEZ implementation. By contrast, we can observe that the main effects for the loan terms remain largely statistically and economically insignificant. Apart from loan maturity, we generally do not observe that the loan terms are predictive of clients’ employment growth, absent the presence of SEZ implementation.

**Table 11:** Quality of access to finance and intensity of treatment effects. The table presents coefficient estimates for a generalized difference-in-differences model measuring the effect of SEZ implementation on client business growth. *DiD estimator* denotes whether a district *d* had SEZ implementation in time *t* (i.e., an interaction on being in the “treatment” group and in the post- “treatment” period). We explore differential treatment effects driven by quality of access to finance through the inclusion of an additional interaction term. The estimation sample includes clients from 115 branches covering the period from 2011 to 2016, of which there are nine branches in the treatment group and 106 in the control. We estimate propensity scores for both treatment and control branch districts on district-level contextual factors driving placement of SEZs. We then apply inverse probability of treatment weighting to create synthetic counterfactual groups for the DiD estimation. All models also include the previously established controls, including client demographics, business characteristics, branch fixed effects, and time fixed effects. Standard errors are clustered at the branch and semester-level and in parentheses. *** p < 0.01, ** p < 0.05, and * p < 0.1

	DV = Abs. employment growth						DV = Rel. (Ln) employment growth					
	(1)	(2)	(3)	(4)	(5)	(6)	(7)	(8)	(9)	(10)	(11)	(12)
*DiD* estimator	− 0.121	0.603 ∗ ∗ ∗	0.193 ∗ ∗	0.429 ∗ ∗ ∗	0.225 ∗ ∗	0.229 ∗ ∗	0.045	0.186 ∗ ∗ ∗	0.115 ∗ ∗ ∗	0.165 ∗ ∗ ∗	0.089 ∗ ∗	0.089 ∗ ∗
	(0.163)	(0.144)	(0.088)	(0.074)	(0.101)	(0.103)	(0.041)	(0.028)	(0.037)	(0.022)	(0.039)	(0.040)
*DiD* estimator = 1 × Loan amount (Ln)	0.059 ∗ ∗ ∗						0.011 ∗ ∗					
	(0.022)						(0.005)					
*DiD* estimator = 1 × Interest rate		− 0.012 ∗ ∗ ∗						− 0.002 ∗ ∗				
		(0.004)						(0.001)				
*DiD* estimator = 1 × Loan duration (yrs.)			0.083 ∗ ∗						0.012			
			(0.032)						(0.014)			
*DiD* estimator = 1 × % Loan amount collateralized				− 0.018 ∗ ∗ ∗						− 0.007 ∗ ∗ ∗		
				(0.006)						(0.001)		
*DiD* estimator = 1 × Loan cycle					0.074 ∗ ∗	0.075 ∗					0.020 ∗	0.021 ∗
					(0.037)	(0.041)					(0.011)	(0.011)
Loan amount (Ln)	0.002					0.057	− 0.005					0.018
	(0.017)					(0.050)	(0.005)					(0.015)
Interest rate		0.001				0.006		0.002				0.003
		(0.004)				(0.012)		(0.001)				(0.004)
Loan duration (yrs.)			− 0.054			− 0.110 ∗ ∗			− 0.024 ∗ ∗			− 0.034 ∗ ∗
			(0.039)			(0.053)			(0.011)			(0.013)
% Loan amount collateralized				− 0.000		− 0.001				0.000		− 0.001
				(0.003)		(0.002)				(0.001)		(0.001)
Loan cycle					− 0.013	− 0.010					0.012	0.013
					(0.025)	(0.025)					(0.008)	(0.008)
Observations	6901	6901	6901	6901	6901	6901	6901	6901	6901	6901	6901	6901
*R*^2^	0.062	0.062	0.062	0.062	0.035	0.036	0.064	0.064	0.065	0.064	0.063	0.065

We also explore differential effects for clients with longer borrower-lender relationships (Hypothesis 4b). A diverse literature has established that length and strength of relationships can be linked with a variety of improved firm outcomes, via improved terms of finance (Kysucky & Norden (2016); Delis et al. (2017)) and greater provision of “soft guidance” to enterprises (Brancati (2015); López-Espinosa et al. (2017); R. N. Banerjee et al. (2021)). We test this in our setting, by using an interaction between the loan cycle of the borrower and the SEZ implementation. In Table 6, columns 5 and 11, we observe positive and significant interactions between the *DiD* estimator and progressive loan cycles. In columns 6 and 12, we also control for the loan terms received by borrowers and show that the improved outcomes for more longer standing borrowers hold irrespective of the loan terms they received. This falls in line with Banerjee et al. (2015a), who observe the largest positive differential effects in terms of business growth from increased access to credit concentrated among clients with more mature intensive margin businesses.

Overall, our combined findings support the hypothesis that SEZ implementation led in turn to a supply-side shock in credit provision, which contributed to enterprise growth. It appears that the onset of SEZs led affected branches to improve key loan terms for borrowers, but did not appear to notably change the amount of or composition of incoming borrowers. The borrowers who received improved loan terms were then better able to grow their businesses, at least in terms of employment.

## Concluding discussion

In this study, we use Cambodia’s special economic zones as a policy shock to analyze how improvements to the local business environment help explain growth outcomes of micro- and small enterprise microfinance clients. By drawing on administrative data from a leading Cambodian microfinance provider, we show that SEZ implementation has positive and economically significant effects on employment growth for MSE clients in SEZ districts compared to those in contextually similar control districts. Key channels explaining the greatest improvements in business growth stemming from the onset of SEZs are linked to both demand-side shocks—expanded markets particularly for agricultural producers—and supply-side shocks—more dynamic labor environments and improved credit terms and conditions.

**Fig. 5:**
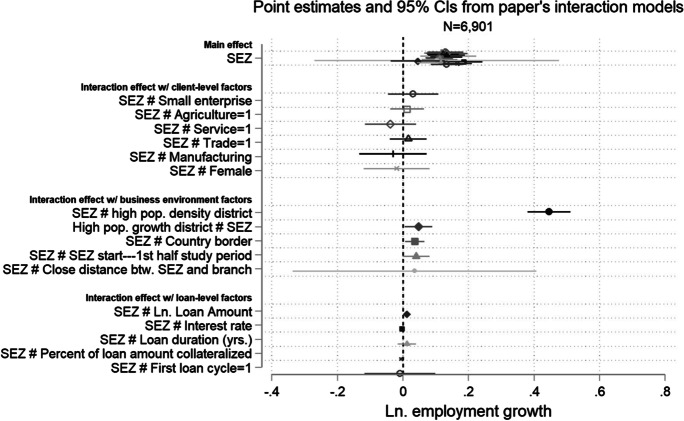
Generalized impact of SEZs and moderators on MSE growth outcomes. This figure summarizes results from the paper’s exploration of contextual factors moderating treatment effect intensity. It provides point estimates and 95% confidence intervals for the main effect (DiD indicator for SEZ implementation) and interaction effect from client, territorial, and loan factors. These correspond to the interaction models described in Section 5.3. The models thus also include the full set of baseline controls and main term. The outcome of interest in the model results here is relative (ln.) employment growth

Figure 5 provides a summary of our main regression results, plotting the coefficients and 95 % confidence intervals from hypothesis tests on our main effect and client- and territorial-level interaction effects. The visualization helps to reiterate that the main effect is economically large—for example, SEZ implementation leads to 10 to 15% greater relative employment growth on average. The effect prevails even after factoring in the interacted client- or business environment moderators. We can further observe statistically and economically meaningful interactions for important additional contextual factors, such as population density or the SEZ’s location and age. Interaction effects are particularly noticeable for the district-level factors. These findings underscore our main point that financial access may not be fulfilling its real potential in low-income settings unless they are coupled with the right opportunities provided by a conducive business environment that reaches micro- and small enterprises.

### Relevance to prior microcredit RCTs

To discuss the broader relevance of our results, we collect and compare data from six prominent microcredit impact studies and use our analysis as a guiding framework to illustrate how related business environment factors help to explain different business outcomes exhibited by their sample borrowers. The studies are Augsburg et al. (2015), Banerjee et al. (2015a), Angelucci et al. (2015), Attanasio et al. (2015), Crépon et al. (2015), and Karlan & Zinman (2011).^23^ Their data provide a rich data source on MSE microfinance client outcomes and collectively capture many variables related to the business environment variables that we used in our analysis. Specifically, we map these data to our analysis framework for measuring an MSE’s business environment so that we can examine the potential channels more closely in the expanded data setting. Furthermore, using these previous studies allows us to increase statistical power and draw on greater variation in analyzing both business outcome indicators and business environment contexts.^24^

Consequently, we extract and combine these data for our analysis. We make indicators on business outcomes across the studies comparable by standardizing their time units, adjusting monetary indicators to PPP-adjusted US dollars, and applying log transformation. In practice, all the studies measured business outcomes in terms of expenses, revenues, and profits and all studies but one also captured changes in business employment. As the studies sometimes captured expenses, revenues, and profits as average estimates for different time periods, we standardize these variables as monthly estimates. Furthermore, since most studies captured monetary values in terms of local currency units, we draw on the World Bank’s World Development Indicator database to convert all values to PPP-adjusted US dollars at the date of each study’s respective baseline survey, in order to make them comparable.

Next, we draw on existing variables from these studies’ data to construct indicators on business environment factors related to the expected causal mechanisms underlying our hypotheses. It is worth noting that some of these go beyond the business environment factors that we are able to directly study in our Cambodian setting. With respect to factors falling more on the supply side, we identify relevant variables and construct district-level proxies for the following: (1) levels of financial and human capital (income, amount of formal credit, and education); (2) quality of local institutions and rule of law (trust in formal institutions, trust in informal institutions, levels of crime); (3) access to better infrastructure (road, electricity, and water infrastructure access); and (4) general business environment indicators (favorable business sentiment, levels of competition, and female empowerment in an area).^25^ With respect to factors falling more on the demand side, we also identify variables capturing (5) “fixed” territorial factors that serve as proxies for market size, including the population density in the study area and whether a location is a country border area.

Finally, for each contextual factor, we run two sample *t*-tests on the combined data from these prior studies to formally test the null hypothesis that it has no significant relationship with a given business indicator. We limit the analysis to “treatment” individuals from the studies, since this would be more in line with the clients from our Cambodian study sample. While the main business metric in our primary analysis is the number of employees, we are able to draw on the wider array of business indicators available from the prior studies to also analyze expenses, revenues, and profits, in order to explore if the factors are associated with more dynamic businesses more generally. Note that the availability of specific contextual and business outcome variables varies by study. Thus, the tests are run on subsets of the data (as detailed in Appendix Table 13).

**Fig. 6:**
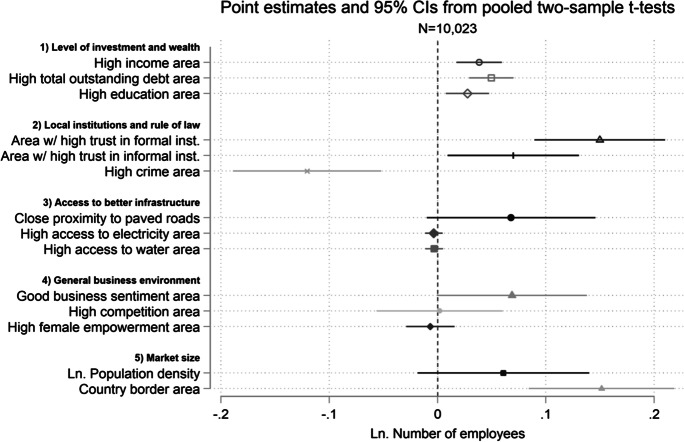
Contextual predictors of business employment from past microcredit RCTs. This figure summarizes results from the paper’s exploration of business environment factors predicting respondent business employment levels in past microcredit RCT studies. We construct variables related to the contextual business environment factors highlighted in our study. After standardizing the data, we run *t*-tests on the null hypothesis that a factor has no significant relationship with log employment at endline. We restrict to the studies’ treatment cohorts and the studies where business employment was recorded (*N* = 10,023). The studies’ datasets differ in availability of specific contextual variables, so the tests are run on relevant subsets of the observations. We use data from (1) Augsburg et al. (2015), (2) Banerjee et al. (2015a), (3) Attanasio et al. (2015), (4) Crépon et al. (2015), and (5) Karlan & Zinman (2011). Appendix Table 13 provides further details on the study contexts and their available contextual and business outcome variables

**Fig. 7:**
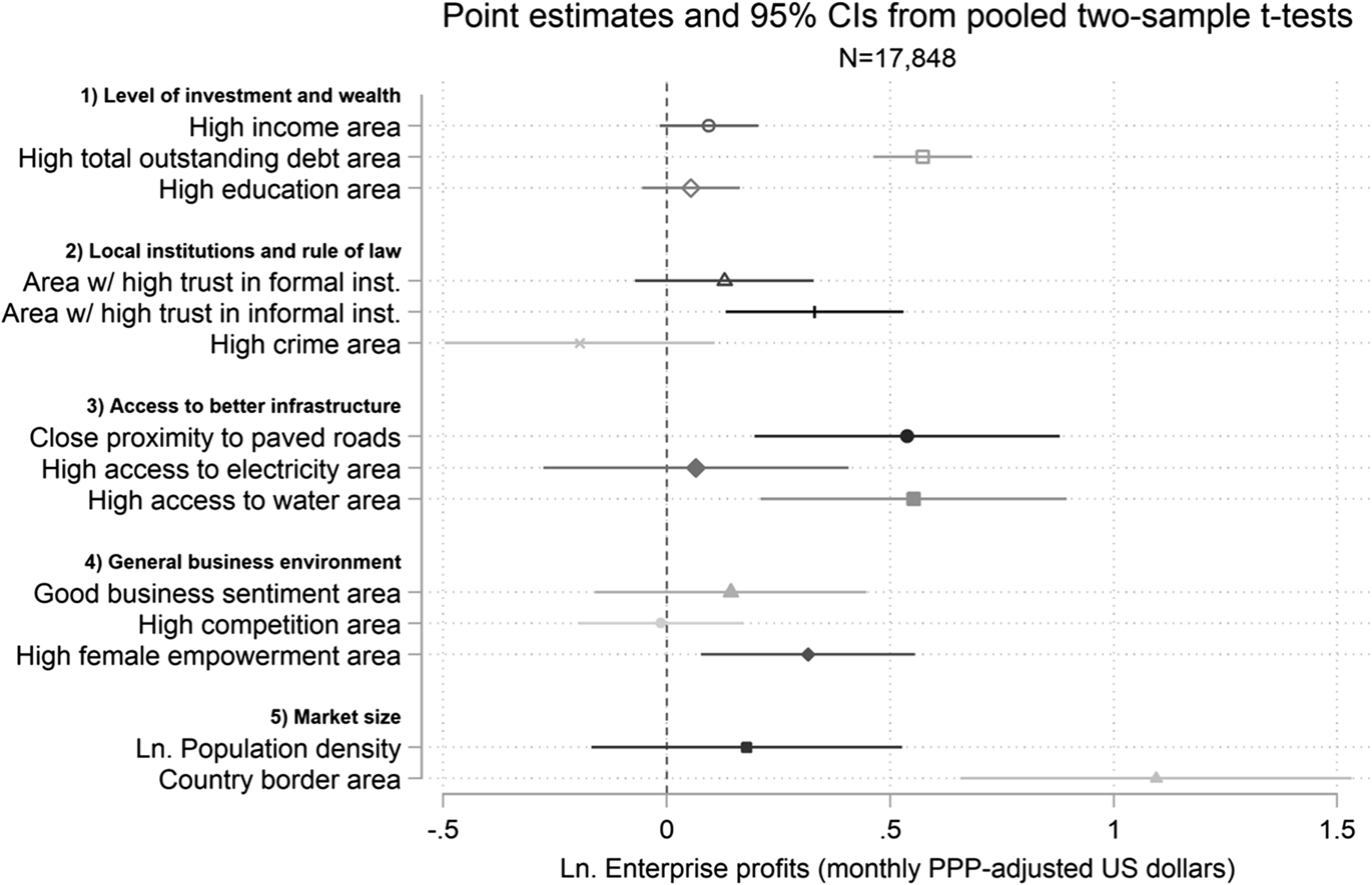
Contextual predictors of business profit from past microcredit RCTs. This figure summarizes results from the paper’s exploration of business environment factors predicting respondents’ business profits in past microcredit RCT studies. The business profits indicator is standardized as monthly PPP-adjusted US dollar values as reported at the time of endline surveys. We construct variables related to the contextual business environment factors highlighted in our study. After standardizing the data, we run *t*-tests on the null hypothesis that a factor has no significant relationship with employment. We restrict our analysis to the studies’ treatment cohorts and the studies where business profits were recorded (*N* = 17,848). The studies’ datasets differ in availability of specific contextual variables, so the tests are run on relevant subsets of the observations. We use data from (1) Augsburg et al. (2015), (2) Banerjee et al. (2015a), (3) Angelucci et al. (2015), (4) Attanasio et al. (2015), (5) Crépon et al. (2015), and (6) Karlan & Zinman (2011). Appendix Table 13 provides further details on the study contexts and their available contextual and business outcome variables

Key insights are depicted in Figs. 6 and 7. Similar to Fig. 5, we plot the coefficients and 95 % confidence intervals from *t*-tests estimating the difference in clients business employment levels and profits across the respective contextual business environment factors. We find strong evidence that these factors play a critical role in influencing clients’ business outcomes across a variety of metrics. We again see that factors that are linked to demand-side aspects of market size and access, such as being located in areas with higher population densities and along country borders, are highly positively related to client business employment levels and profits, consistent with our findings in Section 5.3.3. More importantly, many business environment factors that can be more readily shaped by national and subnational governments also play important and economically meaningful roles. In terms of supply-side inputs, we find strong evidence that higher levels of financial and human capital in local markets are highly positively related to both employment or profits, consistent with our findings in Section 5.3.4. The role of local institutions and rule of law also stands out. For example, we observe that a high level of trust in formal institutions is particularly strongly related to greater employment levels and size of clients’ businesses. This is in line with intuition that entrepreneurs are more willing to risk visibility and grow their businesses when they are less concerned about bureaucratic inefficiencies or malfeasance. Meanwhile, a higher level of trust in informal institutions is disproportionately positively related to higher profits. With respect to infrastructure, we observe that areas with better access to roads, electricity, and water connections exhibit positive associations with profits, but ambiguous associations with employment. Finally, we find mixed evidence with respect to other general business environment indicators. For example, we do not observe any relationship between stated levels of competition and either business outcome. However, areas with better average business sentiment levels and female empowerment index scores show signs of significantly greater employment and profits, respectively.

All in all, the illustrative secondary analysis supports that those factors that can be summarized as capturing key aspects of the “business environment” also matter for the microcredit client MSEs in the context of the prior RCTs.

### Concluding remarks

We emphasize several main messages and policy implications from the combined analyses and discussion. First, looking at the bigger picture, small and micro enterprise investment and growth have long been considered central to the traditional theory of change that links financial access with poverty alleviation. This theory posits that transformative change in businesses is a requisite intermediate outcome that can lead to later improvements on poverty indicators (Beck (2015)). This is relevant whether in terms of partial or general equilibrium effects. Our overall results suggest that the quality of and opportunities provided by local business environments plays an important role in allowing for some MSE microfinance clients to achieve the desired transformative effects. Thus, while microcredit has often been seen as a tool to allow the bottom of the pyramid to bring themselves out of poverty, it ultimately still requires active roles of both national and subnational governments, namely to foster conducive business conditions.

For example, in our Cambodian setting, the national government took proactive steps to study the reasons for its uneven subregional growth and development and develop national policy frameworks for addressing these. Since 1998, it has implemented a “Rectangular Strategy for Growth, Employment, Equity and Efficiency in Cambodia,” which is a comprehensive agenda aimed at improving and building the capacity of public institutions, strengthening good governance, and modernizing the national economic infrastructure, with the objective of promoting inclusive economic growth, generating employment for all citizens, ensuring social equity, enhancing the efficiency of the public sector, and protecting the nation’s natural resources and cultural heritage (Asian Development Bank (2016)). Indeed, the 2005 Law on Special Economic Zones was one of the outcomes of this national-level strategy. Meanwhile, Cambodia has also actively sought to improve public service delivery by bringing government closer to people through ongoing “Decentralization and Deconcentration” (D&D) reforms. Under the D&D reforms, responsibilities for providing government services and implementing many of the initiatives outlined in the “Rectangular Strategy” are being increasingly shifted to subnational administrations, including the commune/Sangkat, district, municipal, or provincial levels of government (Niazi (2011)). To provide a concrete example, local infrastructure development and social service delivery (education and health) have been put mostly under the control of decentralized local authorities.

Second, our paper makes an intuitive but necessary point that the context of past RCT matters in interpreting their results. Clients who had better business environments while also having access to credit appear to be significantly more likely to exhibit growth that could be considered “transformational.” This may paint a different picture concerning the impact that microcredit could have in ideal conditions. It is, however, also important to acknowledge that our results are still in line with existing studies in implying that effects for the *average* client remain small. Like other studies, we find that a key subset of clients drives the majority of business growth. Our findings are consistent with scholars (see, for example, Beck (2015)) who suggest that the promise of microfinance may lie less with trying to promote business transformation en masse among microentrepreneurs and more with allowing transformative growth among opportunity-driven entrepreneurs in order to provide jobs for others. If given the right opportunities, this smaller subset of clients can then have sizeable spillover effects on employment, wages, and income in the broader economy—as also suggested by recent general equilibrium studies (Breza & Kinnan (2021)).

Finally, if the ultimate research objective is to be able to better predict the impact of micro-credit supply in varied settings, then efforts should continue with regard to additional replication studies across more diverse settings—coupled with conscientious data collection in order to capture relevant aspects of those contexts. This would later provide invaluable data to improve our understanding of additional contextual mechanisms that moderate the effects of credit to micro- and small enterprises and move us closer to that objective.

In closing, it is also important to acknowledge several limitations of the paper’s data and analysis that restrict some of the conclusions that can be drawn and point to future research avenues. First, it is worth specifying that the external validity of our results is limited to already financially included enterprises, which may differ from peer enterprises that are not. As we do not have variation in the general use of microcredit in our sample, our results do not directly identify the role of local economic institutions in moderating the treatment effect of microcredit *per se*. Second, the outcome indicators in the paper’s primary analysis are limited to a resource-based measure of enterprise growth—namely, growth in employment—given available data from our partner. Past studies in the empirical enterprise growth literature have documented weaker correlation between resource-based measures and alternative measures for capturing increases in an enterprise’s level of product or service acceptance in the market, such as sales and profits (Wiklund et al. (2009)). Nevertheless, our secondary analysis on data from prior RCT studies helps demonstrate that many of the highlighted contextual factors are also associated with more dynamic businesses when examined using a number of such alternative measures. Finally, the client data used in this study may not capture the full effects of the introduction of a favorable business environment on financially included micro- and small enterprises due to the limited time span that the data cover. Farole (2011), for example, argues that many successful SEZs have required an incubation period, with “the biggest SEZ success stories ... t[aking] at least 5 to 10 years before they began to build momentum.” To the extent that confounding factors are limited in our study areas, repeating the analysis after more time has elapsed could provide informative results.

## Data Availability

The primary data are from a financial service provider and proprietary/not publicly available. The data that support the findings of this study may, however, be made available from the authors upon reasonable request and with extra permission from the provider.

## Appendix 1. Overview of special economic zones

**Table 12:** List of Cambodian special economic zones (2002–2016). This table lists all of the special economic zones opened in Cambodia until the end of 2016. It provides each SEZ’s name, operational start date, district, and province. A “*” denotes an SEZ established in the district during our main study period. Meanwhile, a “**” denotes that there was a prior SEZ established in the district before 2011. To avoid confounding effects from this previous SEZ zone, these respective district branches are omitted from the sample for the paper’s main empirical analysis. The underlying information comes from a database maintained by Open Development Cambodia, which can be found here https://opendevelopmentcambodia.net/profiles/special-economic-zones/

#	Name of SEZ	Start date	District	Province
1	Suoy Chheng SEZ	11/26/2002	Mondol Seima	Koh Kong
2	S.N.C. SEZ	11/26/2002	Prey Nob	Preah Sihanouk
3	Stung Hav SEZ	2/18/2005	Stueng Hav	Preah Sihanouk
4	N.L.C. SEZ	7/15/2005	Svay Teab	Svay Rieng
5	Phnom Penh SEZ	2/20/2006	Posenchey	Phnom Penh
6	Poi Pet O’Neang SEZ	10/7/2005	Ou Chrov	Banteay Meanchey
7	Sihanoukville SEZ 1	10/25/2006	Stueng Hav	Preah Sihanouk
8	Manhattan (Svay Reing) SEZ	8/28/2005	Chantrea	Svay Rieng
9	Oknha Mong SEZ	1/4/2007	Srae Ambel	Koh Kong
10	Kampot SEZ	5/23/2006	Kampot	Kampot
11	Tai Seng Bavet SEZ	1/4/2007	Chantrea	Svay Rieng
12	Goldfame Pak Shun SEZ	1/4/2007	Saang	Kandal
13	Thary Kampong Cham SEZ	6/11/2007	Memot	Kampong Cham
14	Neang Kok Koh Kong SEZ	10/26/2007	Mondol Seima	Koh Kong
15	D&M Bavet SEZ	11/1/2007	Chantrea	Svay Rieng
16	Sihanoukville SEZ 2	9/5/2007	Prey Nob	Preah Sihanouk
17	Kiri Sakor Koh Kong SEZ	12/25/2007	Kiri Sakor	Koh Kong
18	Kampong Saom SEZ	1/6/2009	Stueng Hav	Preah Sihanouk
19	P (SEZ) I C	1/6/2009	Svay Teab	Svay Rieng
20	Sihanoukville Port SEZ	2/8/2008	Preah Sihanouk	Preah Sihanouk
21	MDS Thmorda SEZ	9/30/2010	Veal Veaeng	Pursat
22	KANDAL SEZ*	6/4/2012	Khsach Kandal	Kandal
23	H.K.T. SEZ**	8/15/2012	Prey Nob	Preah Sihanouk
24	Dragon King Bavet SEZ*	8/16/2012	Bavet	Svay Rieng
25	RATANA SEZ**	1/16/2013	Srae Ambel	Koh Kong
26	Try Pheap Ou Ya Dav SEZ*	4/19/2013	Ou Ya Dav	Ratanak Kiri
27	Hi-Park SEZ*	1/16/2013	Bavet	Svay Rieng
28	Shandong Sunshell SEZ*	3/27/2013	Bavet	Svay Rieng
29	Zhong Jian Jin Bian SEZ*	4/19/2013	Kampong Tralach	Kampong Chhnang
30	Sanco Cambo SEZ*	5/3/2013	Poi Pet	Banteay Meanchey
31	Doung Chhiv Phnom Den SEZ*	2/01/2014	Kiri Vong	Takeo
32	Sovannaphum SEZ*	2/11/2014	Kien Svay	Kandal
33	Svay Rieng GIGA SEZ**	7/24/2014	Svay Teab	Svay Rieng
34	Chhak Kampongsaom SEZ**	3/29/2016	Srae Ambel	Koh Kong
35	UBE Snoul SEZ*	6/24/2016	Snuol	Kratie
36	Tian Rui Agricultural Trade SEZ*	6/24/2016	Kong Pisei	Kampong Speu

## Appendix 2. Summary of microcredit RCTs used for secondary analysis

**Table 13:** Study contexts and available data on territorial-level context and entrepreneurial outcomes from past microcredit RCTs. This table summarizes key impact evaluations assessing the effects of microcredit. It adapts Tables 1 and 2 of Banerjee et al. (2015b). We describe relevant aspects of the study and client contexts and available variables on business environments and outcomes. The territorial-level business environment variables are either directly available through the study data or constructed using the primary data. We focus on the following studies as they are considered to be of a high quality, are widely cited, and have provided publicly available data, allowing us to explore the role of relevant contextual factors in explaining heterogeneity in results. The studies are (1) Augsburg et al. (2015), (2) Banerjee et al. (2015a), (3) Angelucci et al. (2015), (4) Attanasio et al. (2015), (5) Crépon et al. (2015), and (6) Karlan & Zinman (2011)

Study:	Bosnia & Herzegovina	India	Mexico	Mongolia	Morocco	Philippines
	(1)	(2)	(3)	(4)	(5)	(6)
*Panel A. Country and study context*						
Country GDP per capita (PPP US dollars)	$8431	$3662	$14,667	$6109	$5455	$1800
Region of study	14 regions nationwide	1 major metropolitan area (Hyderabad)	4 regions in north-central	5 northern regions	11 regions throughout country	Provinces of Rizal, Cavite, and Metro Manila
Location Types	Urban, peri-urban, rural	Urban, peri-urban	Urban, peri-urban, rural	Rural	Peri-urban	Urban, peri-urban, rural
*Panel B. Client-level context*						
Gender of Client	Male, female	Female only	Female only	Female only	Male, female	Male, female
Prior business ownership	51% at baseline	7%	21%	61% at baseline		
Main economic activities	38% agriculture,33% production or trade, and 29% services	24% agriculture, 19% production or trade, 17% services, and 40% other	Not described or captured in the data	34% agriculture or animal husbandry, 37% production or trade, 14% services, 14% other	80–85% Animal husbandry and agriculture, 15–20% non-farm business	0% agriculture, 18% production or trade, 82% services
*Panel C. Business outcomes (variables available (Y/N)?)*						
# of Employees	Y	Y	N	Y	Y	Y
Revenue	Y	Y	Y	Y	Y	Y
Expenses	Y	Y	Y	Y	Y	Y
Profits	Y	Y	Y	Y	Y	Y
Assets	N	Y	Y	N	Y	Y
Investment	N	Y	N	N	Y	N
*Panel D. Territorial-level context at baseline (variables available (Y/N)?)*						
High-income area	Y	Y	Y	Y	Y	Y
High household debt	Y	Y	Y	Y	Y	Y
Area high education area	Y	Y	Y	Y	Y	Y
Area with high trust in formal institutions	N	N	Y	N	N	Y
Area with high trust in informal institutions	N	N	Y	N	N	Y
High crime area	N	N	N	Y	N	N
Close proximity to paved roads	N	N	N	Y	N	N
Access to electricity	N	N	N	Y	Y	N
Access to (piped) water	N	N	N	N	Y	N
Good business sentiment	N	N	N	Y	N	N
Area high competition area	Y	Y	N	N	N	Y
High female empowerment area	N	Y	N	N	Y	N
Population density	N	N	N	Y	N	N
Country border and interior regions	Y	N	Y	Y	N	N

## Appendix 3. Sensitivity tests

In this appendix section, we present results for a number of sensitivity tests that demonstrate that the main results are fairly insensitive to changes in various aspects of the construction of the analysis. This includes alternative methods for using the propensity scores to construct synthetic comparison groups, changing the time unit of analysis, and including or excluding branch districts with SEZs from prior to our sample period from the treatment group. Furthermore, we present suggestive evidence that the main result is unlikely to be driven by differences in client attrition rates between treatment and control districts. It is, however, important to acknowledge that one factor that does appear to influence results (both in this study and in empirical enterprise growth studies more generally) is the treatment of outliers.

### Sensitivity to estimation weighting method

As described in Section 4.3, we choose to apply stability-weighted IPTW in our main analysis. This is to mitigate potential criticisms that certain observations are ending up with unduly large weight, which can increase the variability of the estimated treatment effect (Imbens & Rubin (2015)). To test how sensitive the overall results are to the specific weighting methods used for model estimation, we compare the main analysis results against results when using the normal IPTW method and two other alternatives suggested by Austin & Stuart (2015)—namely, truncated weighting and *combined* stability and truncated weighting. In practice, the normal calculation of IPTW is defined as follows:

5 $$w\left(x\right)\frac{Z}{e}+\frac{\left(1-Z\right)}{\left(1-e\right)},$$

where *Z* denotes treatment status and *e* denotes a district’s propensity score.

Meanwhile, truncated weighting uses thresholds—typically quantiles of the distribution of the weights (for example, the 5th and 95th percentiles)—in which weights that exceed a specified threshold are recoded to that threshold. Strategies may also be combined by truncating after applying stability weighting.

Table 14 provides the results from applying these different weighing methods to our preferred model specification (see Table 5, specifications 2 and 6). Specifically, columns 1 and 5 are the (previously seen) baseline results using stability-weighted IPTW, columns 2 and 6 are results from normal IPTW, columns 3 and 7 use truncated IPTW, and columns 4 and 8 display results from combined truncated and stabilized IPTW. We can observe some slight changes in effect size, but also that our main results remain quite consistent. Moving from stability-weighted IPTW or normal IPTW to truncated IPTW has the largest change in effect size; the *DiD* coefficient is, however, still significant at the 1 % level. We interpret the IPTW and truncated weight results as thus providing some sense with regard to upper and lower bounds, respectively.

### Sensitivity to time unit of analysis

Our analysis in Section 5 uses semester (i.e., 6 months) time units for the difference-in-differences models’ time fixed effects and clustering of standard errors. To demonstrate that the results are fairly insensitive to the choice of time unit of analysis, we rerun the model using annual (i.e., 12 month) time units instead. Table 15 summarizes these results. In comparison to results reported in Table 5, we observe a slight decrease in the statistical significance and economic size of results. Nevertheless, the main effects persist.

**Table 14:** Sensitivity to different estimation methods. This table presents coefficient estimates for a generalized difference-in-differences model measuring the effect of SEZ implementation on absolute and relative (Ln) annualized employment growth. *DiD estimator* denotes whether a district *d* had SEZ implementation in time *t* (i.e., an interaction on being in the “treatment” group and in the post “treatment” period). We explore the sensitivity of main results to the different estimation models. Columns 1 and 5 are our baseline results using stability-weighted IPTW; columns 2 and 6 display results using normal IPTW; columns 3 and 7 provide results using truncated IPTW; columns 4 and 8 provide results using truncated and stability-weighted IPTW. The estimation sample includes clients from 115 branches covering the period from 2011 to 2016, of which there are nine branches in the treatment group and 106 in the control. We estimate propensity scores for both treatment and control branch districts on district-level contextual factors driving placement of SEZs. We then apply inverse probability of treatment weighting to create synthetic counterfactual groups for the DiD estimation. Client controls include gender, household type, and age. Business controls include whether a client’s business is a micro- or small enterprise, economic sector of activity, and whether the client engages in multiple economic activities. Standard errors are clustered at the branch and semester-level and in parentheses. *** p < 0.01, ** p < 0.05, and * p < 0.1

	DV = Abs. employment growth				DV = Rel. (Ln) employment growth			
	(1)	(2)	(3)	(4)	(5)	(6)	(7)	(8)
*DiD* estimator	0.353 ∗ ∗ ∗	0.364 ∗ ∗	0.354 ∗ ∗ ∗	0.382 ∗ ∗ ∗	0.137 ∗ ∗ ∗	0.148 ∗ ∗ ∗	0.129 ∗ ∗ ∗	0.143
	(0.108)	(0.128)	(0.091)	(0.114)	(0.028)	(0.034)	(0.019)	(0.025)
*Additional controls:*								
Branch fixed effects	Yes	Yes	Yes	Yes	Yes	Yes	Yes	Yes
Semester fixed effects	Yes	Yes	Yes	Yes	Yes	Yes	Yes	Yes
Borrower controls	Yes	Yes	Yes	Yes	Yes	Yes	Yes	Yes
Business controls	Yes	Yes	Yes	Yes	Yes	Yes	Yes	Yes
Observations	6901	6901	6901	6901	6901	6901	6901	6901
*R* ^2^	0.062	0.071	0.063	0.064	0.064	0.070	0.063	0.066

**Table 15:** Sensitivity to time unit of analysis. This table presents coefficient estimates for a generalized difference-in-difference model measuring the effect of SEZ implementation on absolute and relative (log) annualized employment growth. *DiD estimator* denotes whether a district *d* had SEZ implementation in time *t* (i.e., an interaction on being in the group and in the post period). The estimation sample includes clients from 115 branches covering the period from 2011 to 2016, from which there are 9 branches falling in the treatment group and 106 falling in the control. We estimate propensity scores for both treatment and control branch districts on district-level factors driving placement of SEZs. We then apply inverse probability of treatment weighting to create synthetic counterfactual groups for the DiD estimation. We estimate different model specifications which include a (a) baseline model with just district and time (semester) fixed effects and our main difference-in-differences estimator, (b) including borrower-level controls, (c) further for business-level characteristics, and (d) also adding in loan characteristics. Client controls include gender, household type, and age. Business controls include whether a client’s business is a micro- or small enterprise, in its first loan cycle or not, economic sector of activity, and whether the client engages in multiple economic activities. Loan characteristics include ln. loan amount (USD), interest rate, loan duration, and percent of loan amount collateralized. Standard errors are clustered at the branch and year-level and in parentheses. *** p < 0.01, ** p < 0.05, and * p < 0.1

	DV = Abs. employment growth				DV = Rel. (Ln) employment growth			
	(1)	(2)	(3)	(4)	(5)	(6)	(7)	(8)
*DiD* estimator	0.371 ∗ ∗ ∗	0.377 ∗ ∗ ∗	0.327 ∗ ∗	0.330 ∗ ∗ ∗	0.140 ∗ ∗ ∗	0.142 ∗ ∗ ∗	0.133 ∗ ∗ ∗	0.132 ∗ ∗ ∗
	(0.055)	(0.072)	(0.074)	(0.071)	(0.014)	(0.011)	(0.016)	(0.025)
*Additional controls:*								
Branch fixed effects	Yes	Yes	Yes	Yes	Yes	Yes	Yes	Yes
Year fixed effects	Yes	Yes	Yes	Yes	Yes	Yes	Yes	Yes
Borrower controls	No	Yes	Yes	Yes	No	Yes	Yes	Yes
Business controls	No	No	Yes	Yes	No	No	Yes	Yes
Loan characteristics	No	No	No	Yes	No	No	No	Yes
Observations	6901	6901	6901	6901	6901	6901	6901	6901
*R* ^2^	0.034	0.035	0.062	0.063	0.053	0.054	0.064	0.066

### Client–lender relationships and attrition

Another concern may be that the study’s main results are biased by differences in attrition rates between the treatment and control districts. This would be problematic if it is also systematically related to differences in the provider’s clients’ business growth. For example, if treatment district clients with the worst performing businesses were disproportionately shut out from obtaining further loans with the provider compared to the control district clients, this could create a positive bias that overstates the “true” treatment effect. There is also an opposite hypothetical scenario where clients in the treatment districts with the best performing businesses simply do not require any further loans and similarly drop out of the sample, but this is of less concern for our main results since this would imply a negative bias—that is, the “true” treatment effect would then be even larger.

To provide supporting evidence that differences in attrition are unlikely to be driving the results, we compare key aspects of client–lender relationships between our treatment and control groups, again adopting a standardized differences approach. Specifically, we calculate the total number of loan cycles, the length of client–lender relationships, and the days between loan cycles for each client. We run descriptive statistics at the unique client level applying our inverse probability of treatment weights. In Table 16, we can observe that the standardized differences between control and treatment clients in terms of their total number of loan cycles, relationship length with the lender, and time between loans do not surpass established thresholds (Imbens & Rubin (2015)). Moreover, cursory observation reveals that the economic size of the differences is rather negligible.

**Table 16:** Summary statistics on client–lender relationships. This table presents summary statistics for client–lender relationships for the treatment and control groups. Specifically, we calculate total number of loan cycles, the length of client–lender relationships, and the days between loan cycles for each client. The estimation sample includes all loans for clients from 115 branches covering the period from 2011 to 2016, of which there are nine branches in SEZ locations and 106 branches in non-SEZ locations. We estimate propensity scores for both SEZ and non-SEZ branch districts on district-level contextual factors driving “treatment”—i.e., placement of SEZs. We then apply inverse probability of treatment weighting to create synthetic counterfactual groups for the DiD estimation. We calculate the normalized difference for a given variable as the difference in means between the treatment and control groups, divided by the square root of half the sum of the treatment and control group variances (Imbens & Rubin (2015)). This provides a scale-invariant measure of the size of differences

	Control		Treatment		Differences	
	Mean	SD	Mean	SD	Differences in Means	Standardized Diff.
Total number of loan cycles	2.71	1.76	2.64	3.57	0.07	0.024
Total relationship length (days)	584.96	396.93	589.60	1084.18	− 4.64	0.006
Days between loans	401.88	277.67	419.74	761.03	− 17.86	0.031
Observations	8838		757		9595	

### Sensitivity to inclusion or exclusion of outliers

A stylized fact from the empirical enterprise growth literature is that growth distributions for enterprises among the general population tend to be heavy-tailed and that the more interesting growth phenomena occur in the tails of the distribution (Coad (2009)). This suggests the need for sensitivity analyses if we are to understand how the presence of outliers affects results in studies on enterprise growth. To do so for this study, we rerun the generalized regressions removing different percentiles of outliers in terms of employment growth from the data sample—specifically, with no outliers winsorized, the top and bottom 1%, top and bottom 2%, and top and bottom 5%.

Table 17 summarizes the results for both absolute and relative employment growth. We can observe that the main result of interest is still fairly consistent after the top and bottom two percentiles have been winsorized. The economic and statistical significance does diminish as an increasing amount of the tails is removed from the sample, roughly halving after the top and bottom five percentiles have been winsorized. While the overall results still stand, the results are in line with the literature in highlighting that the majority of business expansion (and contraction) occurs at the tails of the distribution. Thus, the presence of extreme growth outliers in the sample and the way that the tails of the distribution are treated in the analysis can understandably have an important influence on results.

**Table 17:** Sensitivity to inclusion or exclusion of extreme outliers. This table presents coefficient estimates for a generalized difference-in-differences model measuring the effect of SEZ implementation on client business growth. *DiD estimator* denotes whether a district *d* had SEZ implementation in time *t* (i.e., an interaction on being in the “treatment” group and in the post “treatment” period). We explore the sensitivity of main results to the exclusion of an increasingly larger range of outliers at both the top and the bottom of the distribution in terms of their employment growth. The estimation sample includes clients from 115 branches covering the period from 2011 to 2016, of which there are nine branches in the treatment group and 106 in the control. We estimate propensity scores for both treatment and control branch districts on district-level contextual factors driving placement of SEZs. We then apply inverse probability of treatment weighting to create synthetic counterfactual groups for the DiD estimation. All models also include the previously established controls, including client demographics, business characteristics, branch fixed effects, and time fixed effects. Standard errors are clustered at the branch and semester-level and in parentheses. *** p < 0.01, ** p < 0.05, and * p < 0.1

	DV = Abs. employment growth				DV = Rel. (Ln) employment growth			
	(1)	(2)	(3)	(4)	(5)	(6)	(7)	(8)
	No trim	1 pct. trim	2 pct. trim	5 pct. trim	No trim	1 pct. trim	2 pct. trim	5 pct. trim
*DiD* estimator	0.35* ∗ ∗ ∗ *	0.35* ∗ ∗ ∗ *	0.39* ∗ ∗ *	0.20* ∗ ∗ *	0.14* ∗ ∗ ∗ *	0.13* ∗ ∗ ∗ *	0.11* ∗ ∗ ∗ *	0.08* ∗ ∗ *
	(0.11)	(0.09)	(0.13)	(0.08)	(0.03)	(0.03)	(0.02)	(0.03)
*Additional controls:*								
Branch fixed effects	Yes	Yes	Yes	Yes	Yes	Yes	Yes	Yes
Semester fixed effects	Yes	Yes	Yes	Yes	Yes	Yes	Yes	Yes
Client controls	Yes	Yes	Yes	Yes	Yes	Yes	Yes	Yes
Business controls	Yes	Yes	Yes	Yes	Yes	Yes	Yes	Yes
Observations	6901	6759	6620	6197	6901	6758	6615	6204
*R* ^2^	0.062	0.057	0.051	0.046	0.064	0.056	0.064	0.041

### Sensitivity of results to minimum threshold for growth period

As described in Section 4, we require a minimum threshold of 12 months between loan cycles when calculating clients’ business growth for the paper’s analysis. This is in line with standards from the empirical growth literature, where studies typically calculate growth rates over a minimum period of one year in order to reduce statistical noise and to better account for one-off expansions (Davidsson et al. (2010)). Moreover, this approach takes into consideration the average time between loan applications for clients in our sample (which is slightly above 365 days; see Table 16). Reducing this to a shorter (and less rigorous) threshold does, however, imply a trade-off in terms of increasing the number of sample observations for our regression models. Consequently, to test whether main results are sensitive to the growth period being measured, we reconstruct the main dependent variables restricting ourselves to observations where six or more months have passed between loan applications and rerun the generalized model from Section 5.2.

Table 18 provides a comparison of these results (columns 3 and 4) with our previous results (columns 1 and 2). We observe that the number of observations roughly doubles but that the coefficients become much smaller in economic size and largely insignificant. This suggests that employment growth from SEZ implementation is likely to be nonlinear and indeed may involve sporadic and one-off expansion. Moreover, as intuition would suggest, effects from SEZ implementation are more likely to occur and be observed over longer time scales.

**Table 18:** Sensitivity to growth period. This table presents coefficient estimates for a generalized difference-in-differences model measuring the effect of SEZ implementation on client business growth. *DiD estimator* denotes whether a district *d* had SEZ implementation in time *t* (i.e., an interaction on being in the “treatment” group and in the post “treatment” period). We explore the sensitivity of main results to applying a minimum threshold of 6 months between loan cycles in lieu of 12 months when measuring differences in employment. The estimation sample includes clients from 115 branches covering the period from 2011 to 2016, of which there are nine branches in the treatment group and 106 in the control group. We estimate propensity scores for both treatment and control branch districts on district-level factors driving placement of SEZs. We then apply inverse probability of treatment weighting to create synthetic counterfactual groups for the DiD estimation. All models also include the previously established controls, including client demographics, business characteristics, branch fixed effects, and time fixed effects. Standard errors are clustered at the branch and semester-level and in parentheses. *** p < 0.01, ** p < 0.05, and * p < 0.1

	Min. 12 months btw. loan cycles		Min. 6 months btw. loan cycles	
	(1) Abs	(2) Rel. (Ln)	(3) Abs	(4) Rel. (Ln)
*DiD* estimator = 1	0.353 ∗ ∗ ∗	0.137 ∗ ∗ ∗	0.003	0.002
	(0.100)	(0.001)	(0.047)	(0.020)
*Additional controls:*				
Branch fixed effects	Yes	Yes	Yes	Yes
Semester fixed effects	Yes	Yes	Yes	Yes
Client controls	Yes	Yes	Yes	Yes
Business controls	Yes	Yes	Yes	Yes
Observations	6901	6901	12,756	12,756
*R* ^2^	0.062	0.064	0.028	0.041

### Sensitivity of results to inclusion of branches where SEZs were implemented prior to our sample period

Finally, the regression models in the paper’s main analysis are run on a subset of branches where SEZs were implemented during our data period. We specifically omit clients from branches located in districts with SEZs implemented prior to 2011 to avoid the possibility of confounding effects on business outcomes from previous SEZs. This is based on the expectation that businesses in the districts in which SEZ were implemented prior to the start of our study period may already be experiencing different business growth trends than the rest of the sample due to SEZ implementation, which may influence results.

To explore this, we rerun the model specifications in Table 5 but include all 20 SEZ branch districts. Results are provided in Table 19 and show that the main results essentially hold. We can observe that the economic magnitude of the effect decrease slightly, but that the statistical significance remains unchanged.

**Table 19:** The impact of SEZ implementation on employment growth in clients’ businesses, full SEZ sample. This table presents coefficient estimates for a generalized difference-in-difference model measuring the effect of SEZ implementation on absolute and relative (log) annualized employment growth. *DiD estimator* denotes whether a district *d* had SEZ implementation in time *t* (i.e., an interaction on being in the group and in the post-period). The estimation sample includes clients from 126 branches covering the period from 2011 to 2016, from which there are 20 branches falling in the treatment group and 106 falling in the control. We estimate propensity scores for both treatment and control branch districts on district-level factors driving placement of SEZs. We then apply inverse probability of treatment weighting to create synthetic counterfactual groups for the DiD estimation. We estimate different model specifications which include a (a) baseline model with just district and time (semester) fixed effects and our main difference-in-differences estimator, (b) including borrower-level controls, (c) further for business-level characteristics, and (d) also adding in loan characteristics. Client controls include gender, household type, and age. Business controls include whether a client’s business is a micro- or small enterprise, economic sector of activity, and whether the client engages in multiple economic activities. Loan characteristics include whether it is a first loan cycle or not, ln. loan amount (USD), interest rate, loan duration, and percent of loan amount collateralized. Standard errors are clustered at the branch and semester-level and in parentheses

	DV = Abs. employment growth				DV = Rel. (Ln) employment growth			
	(1)	(2)	(3)	(4)	(5)	(6)	(7)	(8)
*DiD* estimator	0.371 ∗ ∗ ∗	0.379 ∗ ∗ ∗	0.333 ∗ ∗	0.340 ∗ ∗ ∗	0.135 ∗ ∗ ∗	0.138 ∗ ∗ ∗	0.128 ∗ ∗ ∗	0.131 ∗ ∗ ∗
	(0.000)	(0.003)	(0.013)	(0.005)	(0.000)	(0.000)	(0.001)	(0.001)
*Additional controls:*								
Branch fixed effects	Yes	Yes	Yes	Yes	Yes	Yes	Yes	Yes
Semester fixed effects	Yes	Yes	Yes	Yes	Yes	Yes	Yes	Yes
Borrower controls	No	Yes	Yes	Yes	No	Yes	Yes	Yes
Business controls	No	No	Yes	Yes	No	No	Yes	Yes
Loan characteristics	No	No	No	Yes	No	No	No	Yes
Observations	7763	7763	7763	7763	7763	7763	7763	7763
*R* ^2^	0.033	0.034	0.060	0.061	0.049	0.051	0.060	0.061

As an alternative way to see if and to what degree proximity to past SEZs plays a significant role in creating greater impact for the SEZs implemented in our study period, we also construct a variable which captures the distance (by road travel in km) between every treatment and control branch and the nearest SEZs established before 2011. We then introduce this constructed variable—*nearest pre-study period SEZs (in km)*—as an interaction term with our main difference-in-differences estimator and rerun our generalized model (Section 5.2, Eq. 3). Specifically, the coefficient on the interaction term tests whether there are any signs that borrower enterprises in SEZs implemented in our study period that were closer to SEZs implemented in prior periods saw significantly greater (or smaller) growth. The results from this test are reported in Table 20 and, as observed, do not indicate any statistically or economically meaningful interaction effects.

**Table 20:** Distance to prior SEZs and intensity of treatment effect. This table presents coefficient estimates for a generalized difference-in-difference model measuring the effect of SEZ implementation on absolute and relative (log) annualized employment growth. *DiD estimator* denotes whether a district *d* had SEZ implementation in time *t*. To test whether proximity to past SEZs plays a significant role in creating greater impact for the SEZs implemented in our study period, we construct a variable which captures the distance (by road travel in km) between every treatment and control branch and the nearest SEZ established before 2011. We then introduce this constructed variable—*nearest pre-study period SEZs (in km)*—as an interaction term. The estimation sample includes clients from 115 branches covering the period from 2011 to 2016, from which there are 9 branches falling in the treatment group and 106 falling in the control. We estimate propensity scores for both treatment and control branch districts on district-level factors driving placement of SEZs. We then apply inverse probability of treatment weighting to create synthetic counterfactual groups for the DiD estimation. We estimate different model specifications which include a (a) baseline model with just district and time (semester) fixed effects and our main difference-in-differences estimator, (b) including borrower-level controls, (c) further for business-level characteristics, and (d) also adding in loan characteristics. Client controls include gender, household type, and age. Business controls include whether a client’s business is a micro- or small enterprise, economic sector of activity, and whether the client engages in multiple economic activities. Loan characteristics include whether it is a first loan cycle or not, ln. loan amount (USD), interest rate, loan duration, and percent of loan amount collateralized. Standard errors are clustered at the branch and semester-level. *** *p* < 0.01, ** *p* < 0.05, and * *p* < 0.1

	DV = Abs. employment growth				DV = Rel. (Ln) employment growth			
	(1)	(2)	(3)	(4)	(5)	(6)	(7)	(8)
*DiD* estimator	0.357* ∗ ∗ ∗ *	0.373* ∗ ∗ *	0.415* ∗ ∗ *	0.398* ∗ ∗ *	0.113* ∗ ∗ ∗ *	0.120* ∗ ∗ *	0.123* ∗ ∗ *	0.120* ∗ ∗ *
	(0.008)	(0.026)	(0.026)	(0.034)	(0.004)	(0.025)	(0.031)	(0.042)
*DiD* estimator = 1 X Nearest pre-study period SEZ (in km)	0.000	0.000	-0.000	-0.000	0.000	0.000	0.000	0.000
	(0.757)	(0.849)	(0.820)	(0.862)	(0.403)	(0.562)	(0.640)	(0.738)
*Additional controls:*								
Branch fixed effects	Yes	Yes	Yes	Yes	Yes	Yes	Yes	Yes
Semester fixed effects	Yes	Yes	Yes	Yes	Yes	Yes	Yes	Yes
Borrower controls	No	Yes	Yes	Yes	No	Yes	Yes	Yes
Business controls	No	No	Yes	Yes	No	No	Yes	Yes
Loan characteristics	No	No	No	Yes	No	No	No	Yes
Observations	6901	6901	6901	6901	6901	6901	6901	6901
*R* ^2^	0.039	0.040	0.071	0.072	0.060	0.063	0.070	0.073

**Table 21:** Client-level factors affecting intensity of treatment effects, sample restricted to interior, and ocean border districts. The table presents coefficient estimates for a generalized difference-in-differences model measuring the effect of SEZ implementation on client business growth. *DiD estimator* denotes whether a district *d* had SEZ implementation in time *t* (i.e., an interaction on being in the “treatment” group and in the post “treatment” period). We restrict the sample to districts on the country interior or ocean borders. We explore differential treatment effects driven by client-level factors through the inclusion of an additional interaction term. All models also include the previously established controls, including other client demographics (age, dependents), business characteristics (multiple business activities), branch fixed effects, and time fixed effects. Standard errors are clustered at the branch and semester-level and in parentheses. *** p < 0.01, ** p < 0.05, and * p < 0.1

	DV = Abs. employment growth						DV = Rel. (Ln) employment growth					
	(1)	(2)	(3)	(4)	(5)	(6)	(7)	(8)	(9)	(10)	(11)	(12)
*Main effects*												
*DiD* estimator	0.358^∗∗∗^	0.368^∗∗∗^	0.356^∗∗∗^	0.355^∗∗∗^	0.359^∗∗∗^	0.355^∗∗∗^	0.118^∗∗∗^	0.114^∗∗∗^	0.116^∗∗∗^	0.123^∗∗∗^	0.119^∗∗∗^	0.100^∗∗∗^
	(0.075)	(0.078)	(0.072)	(0.090)	(0.075)	(0.076)	(0.024)	(0.025)	(0.023)	(0.028)	(0.024)	(0.028)
Small business	− 1.632^*∗∗∗*^	− 1.632^*∗∗∗*^	− 1.632^*∗∗∗*^	− 1.632^*∗∗∗*^	− 1.632^*∗∗∗*^	− 1.632^*∗∗∗*^	− 0.270^*∗∗∗*^	− 0.270^*∗∗∗*^	− 0.269^*∗∗∗*^	− 0.270^*∗∗∗*^	− 0.270^*∗∗∗*^	− 0.269^*∗∗∗*^
	(0.271)	(0.271)	(0.271)	(0.271)	(0.271)	(0.271)	(0.048)	(0.048)	(0.048)	(0.048)	(0.048)	(0.048)
Agriculture	0.076	0.077	0.076	0.077	0.076	0.076	− 0.021	− 0.022	− 0.021	− 0.022	− 0.021	− 0.020
	(0.649)	(0.649)	(0.649)	(0.649)	(0.649)	(0.649)	(0.237)	(0.237)	(0.237)	(0.237)	(0.237)	(0.237)
Service	0.178	0.178	0.176	0.178	0.178	0.178	− 0.017	− 0.017	− 0.018	− 0.017	− 0.017	− 0.016
	(0.648)	(0.648)	(0.648)	(0.648)	(0.648)	(0.648)	(0.235)	(0.235)	(0.235)	(0.235)	(0.235)	(0.235)
Trade	0.167	0.167	0.167	0.167	0.167	0.167	− 0.010	− 0.010	− 0.011	− 0.010	− 0.010	− 0.009
	(0.642)	(0.642)	(0.642)	(0.642)	(0.642)	(0.642)	(0.235)	(0.235)	(0.235)	(0.235)	(0.235)	(0.235)
Production	0.086	0.086	0.086	0.086	0.087	0.086	− 0.020	− 0.020	− 0.020	− 0.021	− 0.020	− 0.019
	(0.663)	(0.663)	(0.663)	(0.664)	(0.664)	(0.663)	(0.238)	(0.238)	(0.238)	(0.238)	(0.238)	(0.238)
Female	0.002	0.002	0.002	0.001	0.001	0.001	− 0.001	− 0.001	− 0.001	− 0.001	− 0.001	− 0.003
	(0.042)	(0.043)	(0.043)	(0.042)	(0.042)	(0.044)	(0.011)	(0.011)	(0.011)	(0.011)	(0.011)	(0.011)
*Client-level interaction effects*												
Agriculture = 1 × *DiD* estimator = 1		− 0.016 (0.061)						0.006 (0.018)				
Service = 1 × *DiD* estimator = 1			0.079 (0.343)						0.068 (0.122)			
Trade = 1 × *DiD* estimator = 1				0.010 (0.075)						− 0.014 (0.021)		
Production = 1 × *DiD* estimator = 1					− 0.052 (0.136)						− 0.045 (0.043)	
Female × *DiD* estimator = 1						0.007 (0.055)						0.036 (0.034)
Observations	4714	4714	4714	4714	4714	4714	4714	4714	4714	4714	4714	4714
*R* ^2^	0.073	0.073	0.073	0.073	0.073	0.073	0.075	0.075	0.075	0.075	0.075	0.075

## Appendix 4. Details on Cambodian data sample and variables

### Further information on study context

Cambodia is, during our study period, ideal for analyzing the role of subnational business environments in micro- and small enterprise growth, and for several reasons.

First, Cambodia’s economy exhibited general growth and stability at the macro level, which has provided opportunities for both agricultural and nonagricultural businesses. Since early in the new millennium, and apart from a sharp but brief crisis period in 2008,^26^ Cambodia’s economic growth has been among the fastest in Asia, with gross domestic product (GDP) growth averaging 7.9 % from 2000 to 2016.^27^ During this period, its economy benefited from general price stability, with inflation averaging 3.12 %—excluding the crisis year in 2008, and minimal exchange rate fluctuation between the Cambodian riel and the US dollar. This generated social welfare improvements, with a fall in the poverty rate from 50.2 to 17.7 % between 2003 and 2012. This was driven by increases in agricultural prices, wages, and production and by higher incomes and wealth accumulation from self-employment in nonagricultural businesses (Asian Development Bank (2016)).

Second, financial inclusion and access to credit have reached high levels given the country’s development status, allowing us to focus on other constraints to businesses. Domestic credit provided by financial institutions relative to GDP rose from roughly 6 to 71 % from 2000 to 2016, and rates of borrowing from formal institutions reached 27 % in 2017. This has been coupled with high market penetration, with microfinance providers and some leading commercial banks having greatly expanded access for poorer and rural populations. For example, in 2015, Cambodia’s 34 microfinance institutions and seven micro-deposit institutions accelerated their credit growth to 46 % year-on-year and reached a record high of USD 2.9 billion in outstanding loans to over two million borrowers, compared with USD 426 million and 0.9 million borrowers at the end of 2010 ((Asian Development Bank, 2016, 27)). Our research partner is well situated, as it is one of the market leaders in terms of clients served and volume of savings and loans, has nearly nationwide outreach with branches in the vast majority of Cambodia’s 25 provinces and 165 districts, and has a diverse lending portfolio covering micro- through medium-sized enterprises.

Third, development remains uneven across regions and households, with a weak overall business environment cited as a leading barrier to greater growth and opportunities. In 2012, over 90 % of—typically smaller-scale, household-run—nonagricultural enterprises were still estimated to be in the informal sector. Rural and informal enterprises are disproportionately affected by weak public service delivery, limited or lack of access to organized markets, and poor infrastructure, which are thought to be key constraints on their opportunities for growth (Asian Development Bank (2016)). For these reasons, while there have been some general improvements over the past several years, relevant surveys of local entrepreneurs highlight that the overall business environment in Cambodia is still quite poor, albeit with notable heterogeneity across provinces (Malesky (2009)).

### Sample design and extraction

The sample selection and extraction was conducted using the following criteria and steps. First, a target population was defined as any client with their first business loan initiated between January 1, 2011, and December 31, 2014. The extracted loan records for sampled clients cover the period from January 1, 2011, to December 31, 2016, to provide time to track several loan cycles for all clients. Second, from this target population, clients from the research partner’s MIS were then randomly selected. Third, a unique customer identifier was used to link all loan records (cycles) for the sampled clients for extraction. Finally, the data were pooled and transformed into an unbalanced panel dataset at the client-loan level.

### Data and constructed variables

#### Business growth data and variable construction

The research partner’s management information system (MIS) includes a variable capturing the number of employees for a given client’s business at the time of each loan application. Following the literature, we calculate the change in employment levels between loan cycles for each client in terms of both absolute growth and in logarithmic form to capture relative growth. Specifically, our absolute measure captures the raw change in number of employees between the consecutive loan cycles, whereas our relative measure applies a logarithmic transformation of the number of employees followed by first differencing of the log values (which is roughly equivalent to percentage differences). An additional qualification is that, since the dataset is an unbalanced panel (and there may be different lengths of time between consecutive loan observations), the time unit of the growth measure is normalized into an annual growth rate by dividing the relative change between consecutive loans by the amount of time between the application dates of the loan cycles. Furthermore, to reduce the chance that annualized rates may exaggerate growth if two consecutive loans for a client were somehow received within a short time period, we apply an additional criterion that a minimum of 12 months must pass between consecutive loans for the associated growth observation to be included in the data sample. To be specific, those flagged as not meeting these criteria are excluded from the analyses. This is in line with standards from the empirical growth literature, where studies typically calculate growth rates over a minimum period of 1 year in order to reduce statistical noise and to better account for one-off expansions (Davidsson et al. (2010)).

In practice, our initial data sample included records for 11,653 unique borrowers. Among these, 2867 clients do not have their consecutive loan cycles meeting the 12 month criteria and are dropped. There are also 2058 clients who only have 1 loan cycle throughout the entire sample period, where we are unable to calculate growth rates and are also by default not included in the analyses.

#### SEZ data and variable construction

The paper’s main explanatory variable is a dummy indicator constructed at the branch district and time-period level that equals 1 if there is an SEZ implemented in a district in a time period and 0 otherwise. The underlying information for its construction comes from a database maintained by Open Development Cambodia (ODC), which can be found at https://opendevelopmentcambo.net/profiles/special-economic-zones/. This dummy indicator is then mapped to the MIS file at the branch district and time level. In other words, it denotes whether a client in a given branch and time period was in a district location with an implemented SEZ.

We construct several additional variables to provide further context on the SEZs. First, we use an administrative map of Cambodia to construct a dummy indicator for whether the district of a given SEZ is located on an ocean border or on a country border. Source www.stat.go.jp/info/meetings/cambodia/pdf/00dis_mp.pdf. Second, we use a map from the Greater Mekong Subregion Information Portal to construct a dummy indicator for whether a given district is along the Central, Southern, or Southern Coastal GMS corridors. Source http://portal.gms-eoc.org/maps?cmbIndicatorMapType=archive&cmbIndicatorTheme=29&cmbIndicatorMap=24

#### District context variables

Data on contextual characteristics of districts are drawn from the 1998 and 2008 Cambodian General Population Surveys, which were conducted by the Cambodian National Institute of Statistics. These data are available at http://celade.cepal.org/redkhm/census/khm1998/ and http://celade.cepal.org/redkhm/census/khm2008/. We use these sources to obtain information on district population size, population growth rate, land area in square kilometers, and population density. With the exception of population growth, which relies on calculating annualized change from 1998, the figures used in the paper are from the 2008 census, which should provide a fairly accurate depiction of district characteristics at the start of our study period, 2011.

#### Control variable data and construction

Data for the client-, business-, and loan-level controls used in the paper’s analysis are drawn from the MIS sample. It is also worth mentioning that to facilitate interpretation of monetary figures, any values in Cambodian riel—for example, loan amount, and initial assets—are converted into US dollars using the average annual exchange rates for the year of a given loan application, and these values are then adjusted for inflation (that is, deflated) to 2011 levels. The main control variables are:

- *Female*: a dummy indicator for whether a client is female (= 1) or male (= 0).
- *Dependents*: a dummy indicator for whether a client is married (= 1) or single (= 0).
- *Age*: the age of the applicant recorded at the time of the loan application.
- *Economic*_*sector*: The MIS data contains a variable that captures more than 150 types of business activity. We use this information to aggregate the business activities into five broader categories: *Agriculture*, *Service*, *Trade*, *Manufacturing*, and *Other*, which represent dummy indicators set equal to 1 when a client’s business activity falls into a given category, and 0 otherwise.
- *Multiple*_*activities*: to capture whether a client engaged in multiple types of business activity, we further construct a dummy indicator for whether a given client’s *Economic*_*sector* ever changes across loans during the study period. (Data exploration reveals that between 15 and 20% of clients took out loans that finances activities in more than one economic sector.)
- *Small*_*enterprise*: a dummy indicator for whether a client’s business was classified as a small enterprise (= 1) or a microenterprise (= 0) at the time of their first loan cycle with the research partner. We use IFC’s classification scheme, which categorizes microenterprises as those with less than 10 employees and small enterprises as those with between 11 and 50 employees.
- *Loan*_*cycle*: The MIS has a variable that captures the loan cycle number for a given client. We use this to serve as a proxy for shorter- or longer-term (and arguably stronger) lending relationships. We also use this to construct a dummy variable that is set equal to 1 if it is a borrower’s first loan cycle and 0 otherwise, which is used as a control variable for some model specifications.
- *Loan*_*amount*: a continuous variable with the loan amount in USD. We convert this to logarithmic form for some of our analyses.
- *Interest*_*rate*: a continuous variable with the interest rate for a given loan in annualized percentage rate. We convert this to logarithmic form for some of our analyses.
- *Loan*_*duration*: the MIS has variables for the date of loan disbursement and the date of loan maturity. We use these to construct a continuous variable with the total duration of the loan, which we depict in years.
- *Percent*_*loan*_*amount*_*collateralized*: a continuous variable which is constructed as the total collateral amount over the total loan amount.

## Footnotes

1 Indeed, Cambodia’s government promoted SEZs as a way to increase financing for sectors such as garments in the country interior and agricultural production in the hinterlands bordering Thailand and Vietnam. To a large extent, SEZs sought to capitalize on their competitive advantages in terms of lower relative wages and the availability of fertile and cheaper land (Warr & Menon (2016)).

2 The consistency of the finding across both absolute and relative growth measures is important since the enterprise growth literature has established that absolute growth indicators tend to be biased toward highlighting growth in already larger businesses (see, for example, Daunfeldt et al. (2014)).

3 There are also a few recent empirical studies on dynamic effects of SEZs, conducted by Wang (2013), Ciz˙kowicz et al. (2016), and Alkon (2018). With the exception of Ciz˙kowicz et al. (2016), who look at firm-level effects in the context of Poland, these explore territorial-level outcomes.

4 Attanasio et al. (2015) and A. Banerjee, Duflo, et al. (2015) are currently the longest-term studies, with 42- and 43-month follow-up surveys, respectively.

5 The number of SEZs has grown from 176 in 47 countries in the 1980s to 3500 in 130 countries in 2008 (Akinci & Crittle (2008)).

6 As one example, scholars supporting the “strategic fit” perspective have found that entrepreneurial orientation is associated with better performance among small firms operating in hostile environments, but not among those operating in more benign environments (Covin & Slevin (1989); Prajogo (2016)).

7 Hsieh and Olken (2014) provide some empirical evidence supporting this “constrained large firm” model, by using detailed comprehensive firm data from India, Indonesia, and Mexico and finding that the average marginal product of labor and capital is significantly lower in smaller firms.

8 For example, recent reports note that while Cambodia exports agricultural goods to over 90 countries, Vietnam accounts for 64.11% of the exports (3.8 million tonnes), followed by Thailand at 21.49% (1.3 million tonnes) and China at 9.69% (around 575,000 tonnes). Without providing figures, the Minister of Agriculture, Forestry, and Fisheries has reported that sales of agricultural merchandise to the top three buyers “have in- creased”, on the back of rising production in Cambodia and improved transportation systems to neighboring countries. See, for example, https://www.phnompenhpost.com/business/agriculture-exports-near-6m-tonnes-jan-sept.

9 Furthermore, surveys conducted by Warr and Menon (2016) on SEZ-located firms reveal that employment conditions, including average wages, are considered quite good by Cambodian standards.

10 The sample excludes the provider’s medium-sized enterprises and group loans. The provider’s annual reports indicate that medium enterprise loans comprise a small portion of their loan portfolio in terms of numbers of clients—ranging from 4 to 5% of their clients between 2011 and 2016.

11 We also explored growth in client assets as another possible dependent variable. It is ambiguous, however, in the sense that it encompasses a client’s combined assets—that is, both household and business. As such, it may not be necessarily a direct measure of changes in a client’s business per se, particularly for microenterprises, who are less likely to have separated assets. We ultimately focus on employment size since it is a direct and unambiguous proxy for business growth.

12 Daunfeldt et al. (2014) show that high-growth firms defined in relative terms tend to be smaller than those that are fast growing in absolute terms; that is, absolute growth measures are biased toward larger firms, and vice versa.

13 It is worth acknowledging that the outcome indicators in the paper’s primary analysis are thus limited to a resource-based measure of enterprise growth—namely, growth in employment—given the available data from our partner. Past studies in the empirical enterprise growth literature have documented weaker correlation between resource-based measures and alternative measures for capturing increases in an enterprise’s level of product or service acceptance in the market, such as sales and profits (Wiklund et al. (2009)), making it useful to examine these separately where possible. To this end, our secondary analysis on data from prior RCT studies in Section 6.1 helps demonstrate that many of the highlighted contextual factors are also associated with more dynamic businesses when examined using a number of additional relevant business indicators, including profits.

14 https://opendevelopmentcambodia.net/profiles/special-economic-zones/

15 Data obtained from http://celade.cepal.org/redkhm/census/khm1998// and http://celade.cepal.org/redkhm/census/khm2008/

16 Cambodia has four tiers of administration: (1) 24 provinces and the special administrative unit Phnom Penh (25 de facto provinces); (2) 171 districts; (3) 1,621 communes; and (4) 14,073 villages (figures from the 2008 census).

17 That is, the districts with SEZs implemented prior to the start of our study period may already have different—and arguably more positive—business growth trends than the rest of the sample due to the implementation. To explore this, we also run the main models including all twenty branches and provide results in Appendix 3.

18 For example, treatment observations with a high probability of receiving treatment provide less useful counterfactual information and are thus given relatively lower weights in the calculation of average treatment effects. Meanwhile, treatment observations that have a low probability of receiving treatment—but did get treated—are unusual and more informative of how treatment would affect subjects with a low probability of receiving it—that is, they have characteristics that are more akin to control subjects. Therefore, they are given greater weight. Following the same idea, if a control subject has a high probability of receiving treatment, it is an informative indicator of how subjects in the treatment group would behave if they were in the control group, and thus greater weight is placed on these highly informative control subjects.

19 The most common potential criticism of IPTW is that treated observations with a propensity score very close to 0 or control observations with a propensity score very close to 1 can end up with unduly large weight, which can increase the variability of the estimated treatment effect (Imbens & Rubin (2015)). To help mitigate this issue, Austin and Stuart (2015) suggest the alternative of using stabilized weighting and truncated weighting. We conduct sensitivity tests using these different methods in Appendix 3 to demonstrate that main results remain regardless of specific weighting method used.

20 As Imbens & Rubin (2015) highlight, this approach provides a scale-invariant measure of the size of mean differences, and they use this with propensity score matching to demonstrate that differences of 1.0 or more are problematic in terms of giving results similar to an experiment, while differences of 0.25 or less are indicative of good balance. For further rationale for using standard mean differences over *t*-tests, see Stuart (2010).

21 We have also run the event study and main regression models using an annual time unit of analysis, and results are consistent.

22 For completeness, we also tested inclusion of province–semester interactions and also explore whether results are primarily driven by individual SEZs and clients in their corresponding branches. With respect to the former, the inclusion of province–semester interactions does lead to some changes in the economic size of results. It is worth noting, however, that their inclusion increases both the statistical and the economic size of results rather than diminishes them. Moreover, the results would generally support the underlying argument that differences in subnational environments ultimately play a primary role in influencing clients’ business growth outcomes. With respect to the latter, there are some indications that clients in the Poi Pet district branch exhibited stronger growth than average and, conversely, that clients in the Kampong Tralach district branch exhibited weaker growth than average. However, the main effect remains whether these particular branches are included in the sample or not.

23 All of these studies feature in an influential summary article by A. Banerjee, Karlan, and Zinman (2015), with the exception of Karlan and Zinman (2011), which we add because its replication data include a wide set of contextual variables. An additional study by Tarozzi et al. (2015) also featured in the A. Banerjee, Karlan, and Zinman (2015) special issue, but we were unable to locate its replication data.

24 See Appendix Table 13 for an overview of the specific studies and the types of business outcome and environment data they contain. In practice, the combined data yield 35,342 observations. Out of these, 18,979 are treatment observations used for the analysis; they cover 285 country subregions.

25 To operationalize these business environment variables in a way consistent with the studies and our own Cambodian data, we calculate country subregional averages. We then construct dummy variables that flag country subregions that are in the top tercile with respect to a given business environment factor.
